# Supplementary material for: The relative binding position of Nck and Grb2 adaptors impacts actin-based motility of Vaccinia virus
Source: eLife. 2022 Jul 7;11:e74655. doi: 10.7554/eLife.74655 (PMC9333988; doi:10.7554/eLife.74655)
Supplement: Figure 2—source data 1. [file elife-74655-fig2-data1.zip › Figure 2 - source data 1/Figure 2_stats summary table.docx]

| *Figure* | *Measurement* | *Conditions* | *Test* | *p value* | *95% CI lo* | *95% CI hi* |
| --- | --- | --- | --- | --- | --- | --- |
| Fig2B | % virus w/ tails | A36 N-G vs A36 G-N | Welch’s t | 0.80926575 | -8.58 | 10.05 |
| Fig2B | Tail length | A36 N-G vs A36 G-N | Welch’s t | 1.70E-03 | -2.32 | -1.07 |
| Fig2C | Virus speed | A36 N-G vs A36 G-N | Welch’s t | 0.01622998 | -0.12 | -0.02 |
| Fig2D | Plaque size | A36 N-G vs A36 G-N | Welch’s t | 0.00570193 | -0.45 | -0.15 |

* multiple comparisons tests
