# Supplementary material for: The relative binding position of Nck and Grb2 adaptors impacts actin-based motility of Vaccinia virus
Source: eLife. 2022 Jul 7;11:e74655. doi: 10.7554/eLife.74655 (PMC9333988; doi:10.7554/eLife.74655)
Supplement: Figure 2—figure supplement 1—source data 1. [file elife-74655-fig2-figsupp1-data1.zip › Figure 2 - supplement 1 - source data 1/Figure 2 - supplement 1_stats summary table.docx]

| *Figure* | *Measurement* | *Conditions* | *Test* | *p value* | *95% CI lo* | *95% CI hi* |
| --- | --- | --- | --- | --- | --- | --- |
| Fig2-supp1A | % virus w/ tails | A36 N-G vs A36 N-X | Dunnett’s* | 0.6993 | -3.643 | 7.018 |
| Fig2-supp1A | % virus w/ tails | A36 N-G vs A36 X-G | Dunnett’s* | <0.0001 | 11.72 | 22.38 |
| Fig2-supp1A | % virus w/ tails | A36 N-G vs A36 X-X | Dunnett’s* | <0.0001 | 14.62 | 25.28 |
| Fig2-supp1A | Tail length | A36 N-G vs A36 N-X | Dunnett’s* | 0.0022 | 0.6130 | 2.154 |
| Fig2-supp1A | Tail length | A36 N-G vs A36 X-G | Dunnett’s* | <0.0001 | 1.473 | 3.014 |
| Fig2-supp1A | Tail length | A36 N-G vs A36 X-X | Dunnett’s* | <0.0001 | 3.236 | 4.777 |
| Fig2-supp1B | % virus w/ tails | A36 N-X vs A36 X-N | Welch’s t | 0.180999 | -1.78 | 6.13 |
| Fig2-supp1B | Tail length | A36 N-X vs A36 X-N | Welch’s t | 0.18949283 | -1.35 | 0.39 |
| Fig2-supp1C | Plaque size | A36 G-N clone 1 vs 2 | Welch’s t | 0.57525568 | -0.26 | 0.41 |

* multiple comparisons tests
