# Supplementary material for: The relative binding position of Nck and Grb2 adaptors impacts actin-based motility of Vaccinia virus
Source: eLife. 2022 Jul 7;11:e74655. doi: 10.7554/eLife.74655 (PMC9333988; doi:10.7554/eLife.74655)
Supplement: Figure 2—figure supplement 2—source data 1. [file elife-74655-fig2-figsupp2-data1.zip › Figure 2 - supplement 2 - source data 1/Figure 2 - supplement 2_stats summary table.docx]

| *Figure* | *Measurement* | *Conditions* | *Test* | *p value* | *95% CI lo* | *95% CI hi* |
| --- | --- | --- | --- | --- | --- | --- |
| Fig2-supp2A | % virus w/ tails | A36 N-G vs A36 N-N | Tukey’s* | 0.0603 | -13.69 | 0.3448 |
| Fig2-supp2A | % virus w/ tails | A36 N-N vs A36 G-G | Tukey’s* | <0.0001 | 19.31 | 33.34 |
| Fig2-supp2A | Tail length | A36 N-G vs A36 N-N | Tukey’s* | 0.3164 | -0.5598 | 1.686 |
| Fig2-supp2A | Tail length | A36 N-N vs A36 G-G | Tukey’s* | 0.0058 | 0.9076 | 3.419 |
| Fig2-supp2B | % virus w/ tails | A36 N-G 3x vs A36 G-N 3x | Welch’s t | 0.43878577 | -18.18 | 10.96 |
| Fig2-supp2B | Tail length | A36 N-G 3x vs A36 G-N 3x | Welch’s t | 0.03873604 | -2.17 | -0.12 |

* multiple comparisons tests
