# Supplementary material for: The relative binding position of Nck and Grb2 adaptors impacts actin-based motility of Vaccinia virus
Source: eLife. 2022 Jul 7;11:e74655. doi: 10.7554/eLife.74655 (PMC9333988; doi:10.7554/eLife.74655)
Supplement: Figure 3—source data 1. [file elife-74655-fig3-data1.zip › Figure 3 - source data 1/Figure 3_stats summary table.docx]

| *Figure* | *Measurement* | *Conditions* | *Test* | *p value* | *95% CI lo* | *95% CI hi* |
| --- | --- | --- | --- | --- | --- | --- |
| Fig3C | % virus w/ tails | N-WASP +/+ vs -/- | Welch’s t | 0.01762321 | -35.1 | -9.36 |
| Fig3D | % virus w/ tails | p14 N-G vs Y96F | Dunnett’s* | 0.0009 | 11.02 | 31.81 |
| Fig3D | % virus w/ tails | p14 N-G vs Y100F | Dunnett’s* | 0.3844 | -5.144 | 15.65 |
| Fig3D | % virus w/ tails | p14 N-G vs Y116F | Dunnett’s* | 0.0075 | 4.833 | 25.62 |
| Fig3D | Tail length | p14 N-G vs Y96F | Dunnett’s* | 0.0010 | 0.7896 | 2.304 |
| Fig3D | Tail length | p14 N-G vs Y100F | Dunnett’s* | 0.6587 | -1.014 | 0.5004 |
| Fig3D | Tail length | p14 N-G vs Y116F | Dunnett’s* | 0.0077 | 0.3462 | 1.860 |
| Fig3E | % virus w/ tails | p14 N-G vs Y96,116F | Welch’s t | 2.04E-04 | -23.56 | -20.84 |

* multiple comparisons tests
