# Supplementary figures and images for: The relative binding position of Nck and Grb2 adaptors impacts actin-based motility of Vaccinia virus
Source: eLife. 2022 Jul 7;11:e74655. doi: 10.7554/eLife.74655 (PMC9333988; doi:10.7554/eLife.74655)

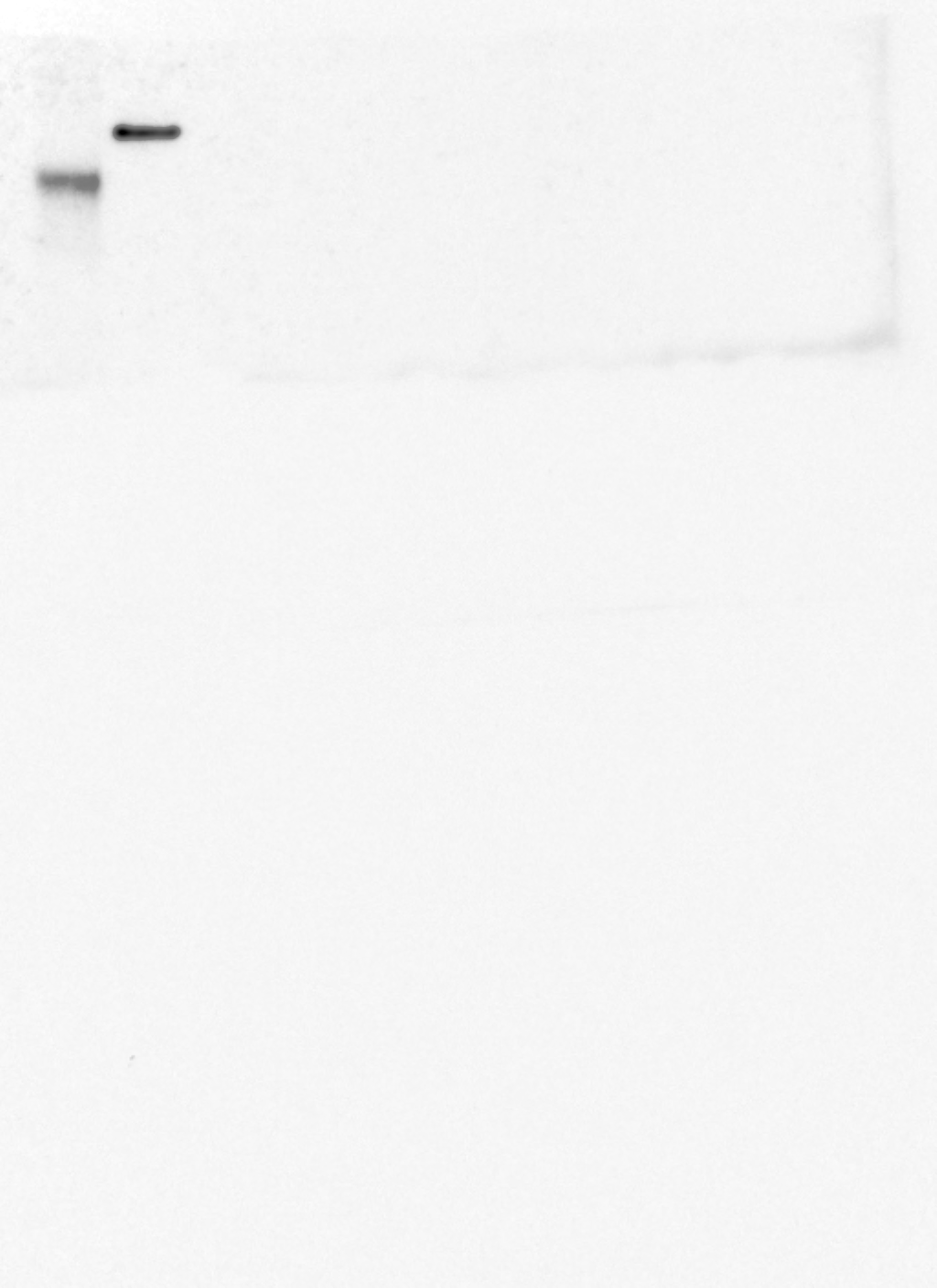

Supplement: Figure 3—source data 1. [file elife-74655-fig3-data1.zip › Figure 3 - source data 1/Fig3F blots/Fig3F_Grb2 blot_raw.tif]

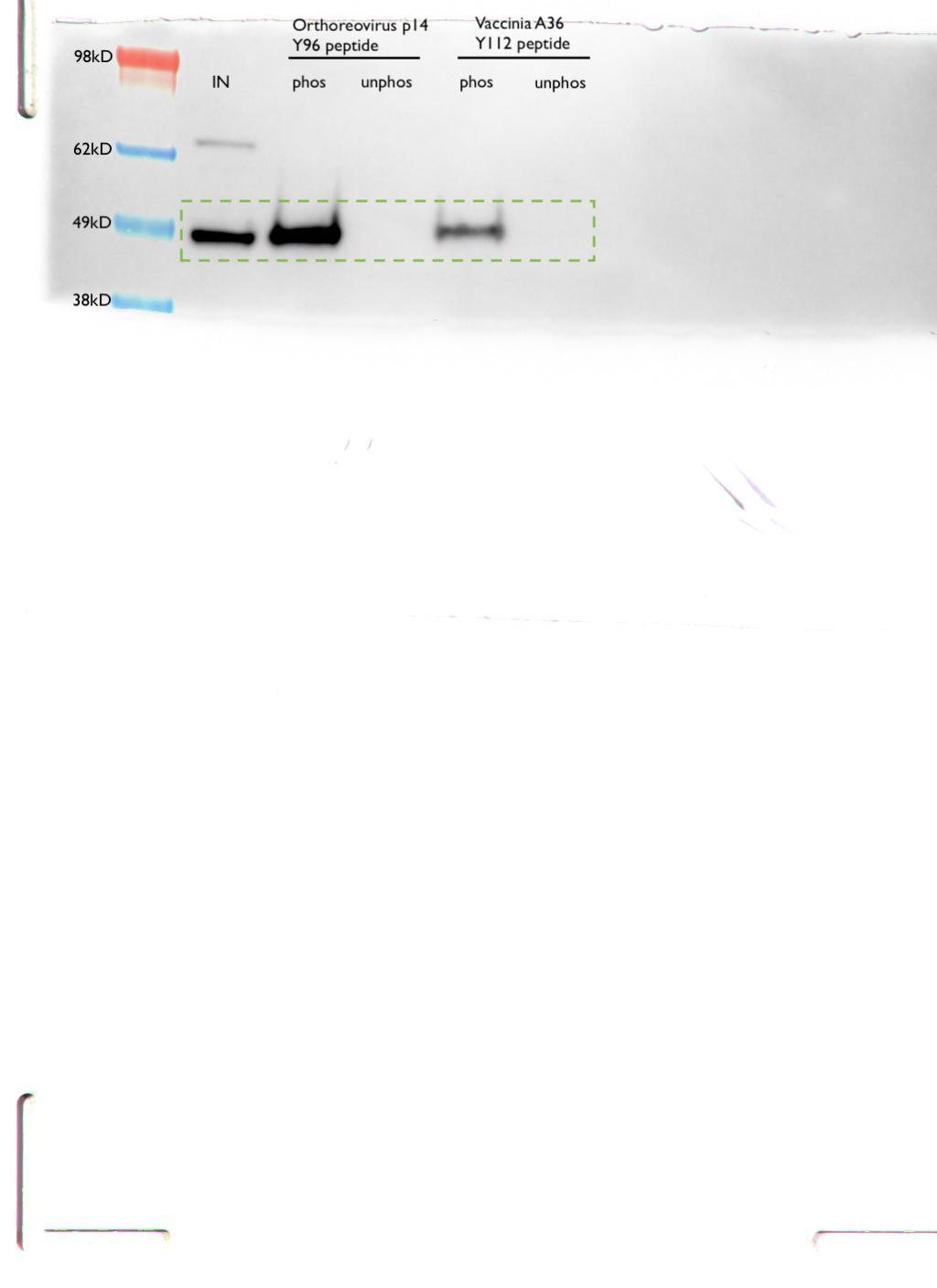

Supplement: Figure 3—source data 1. [file elife-74655-fig3-data1.zip › Figure 3 - source data 1/Fig3F blots/Fig3F_Nck blot_annotated.jpg]

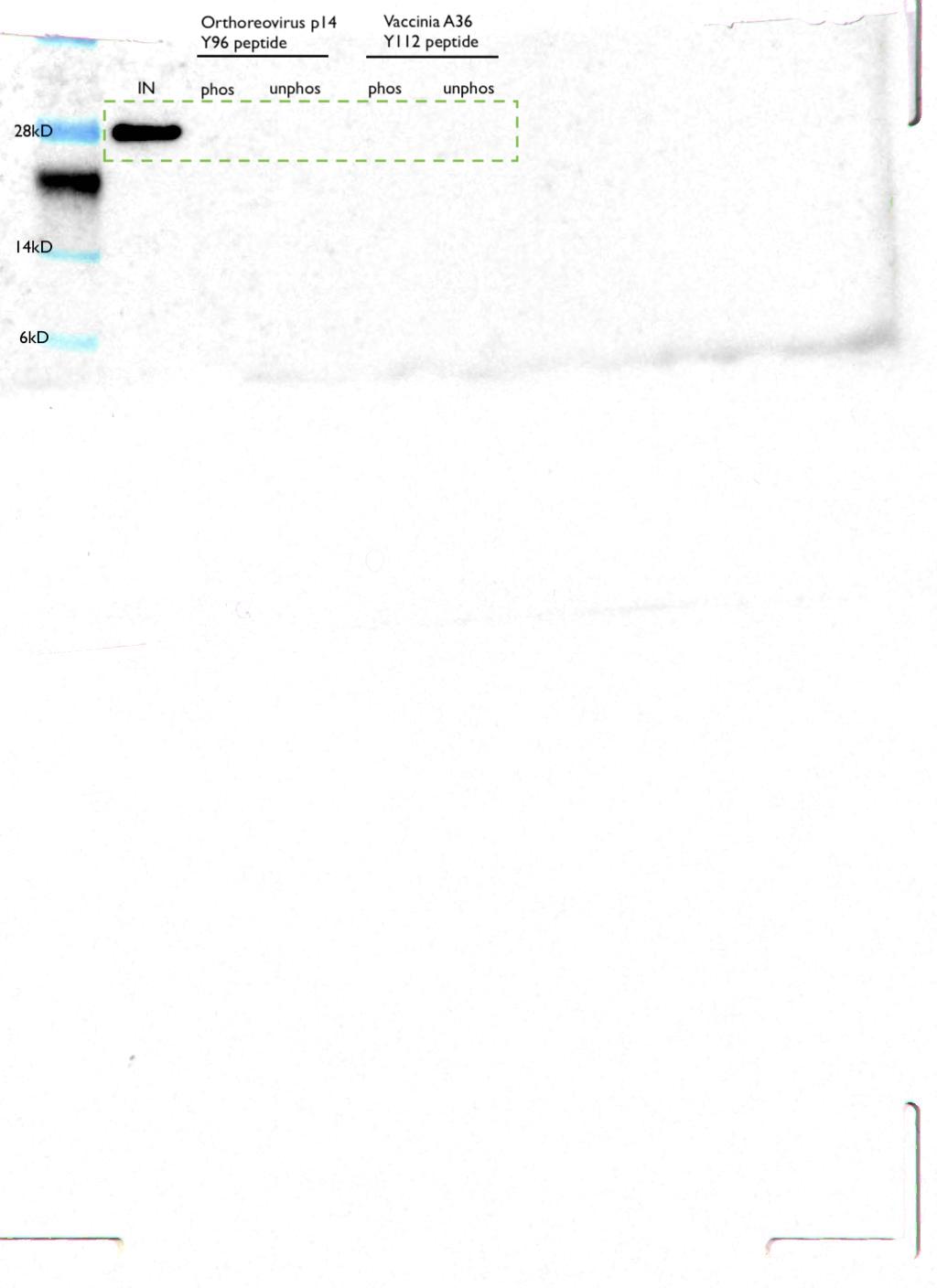

Supplement: Figure 3—source data 1. [file elife-74655-fig3-data1.zip › Figure 3 - source data 1/Fig3F blots/Fig3F_Grb2 blot_annotated.jpg]

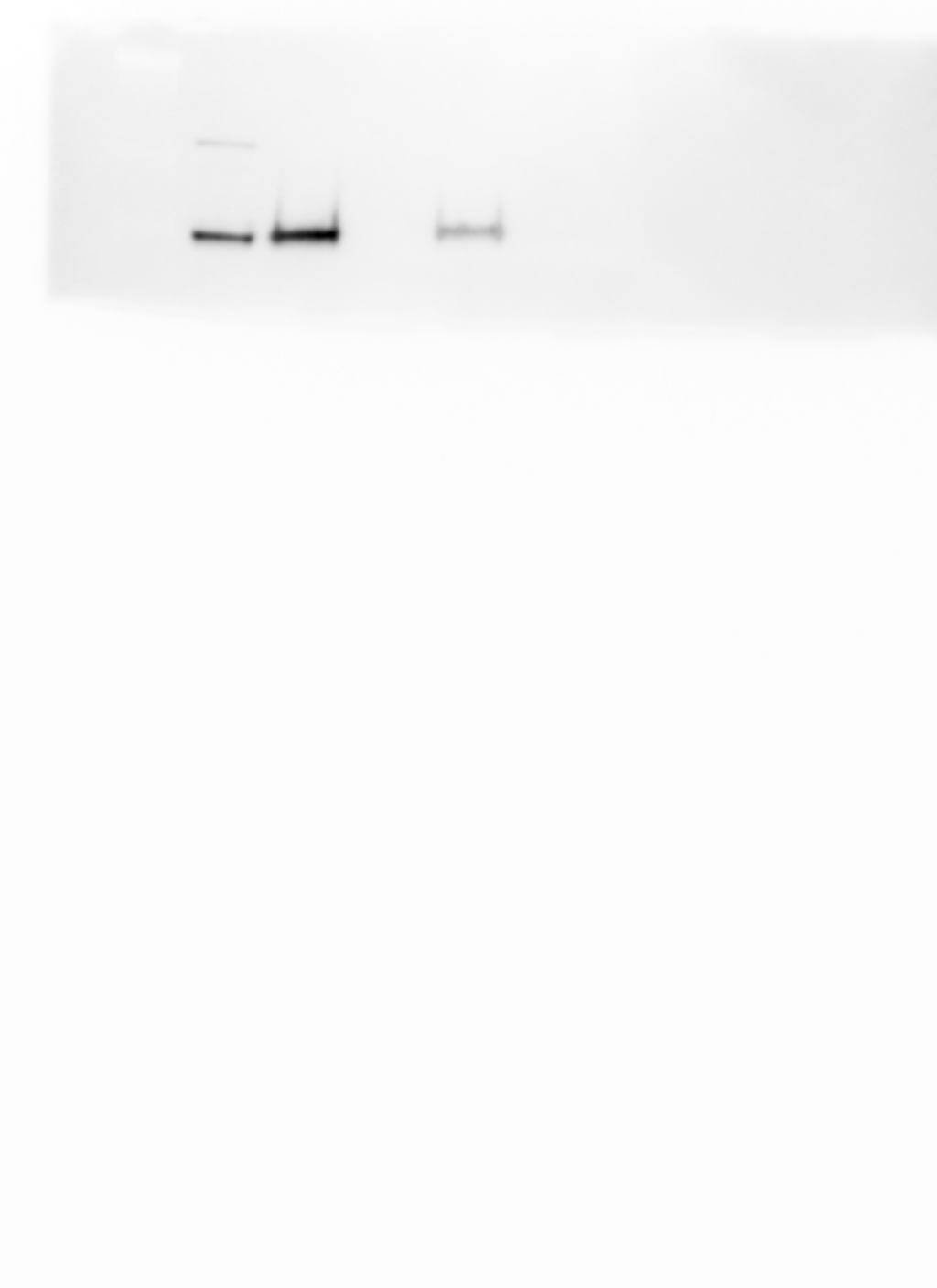

Supplement: Figure 3—source data 1. [file elife-74655-fig3-data1.zip › Figure 3 - source data 1/Fig3F blots/Fig3F_Nck blot_raw.tif]

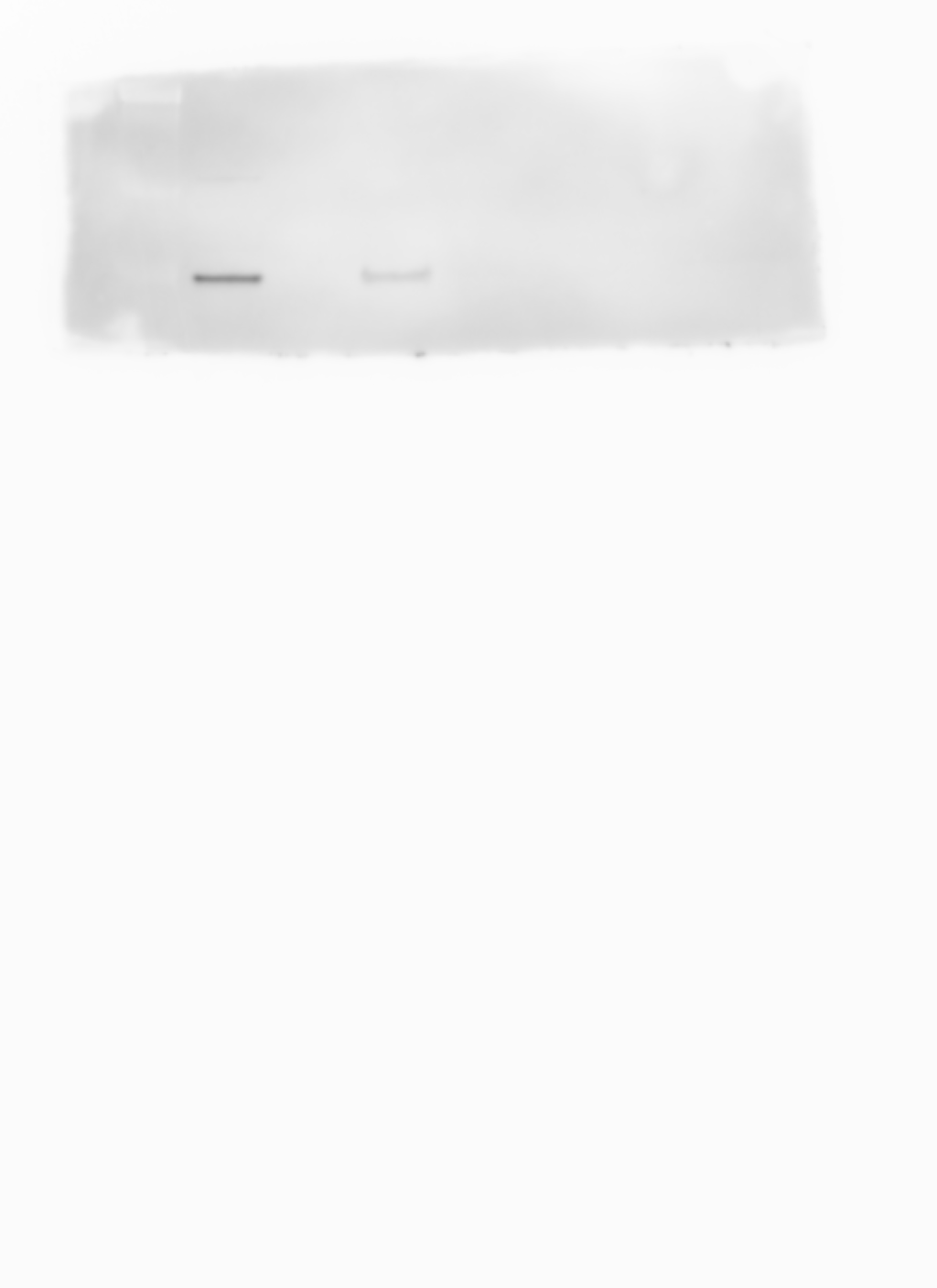

Supplement: Figure 3—figure supplement 1—source data 1. [file elife-74655-fig3-figsupp1-data1.zip › Figure 3 - supplement 1 - source data 1/Fig3-suppl1B blots/Fig3-suppl1B_Nck blot_raw.tif]

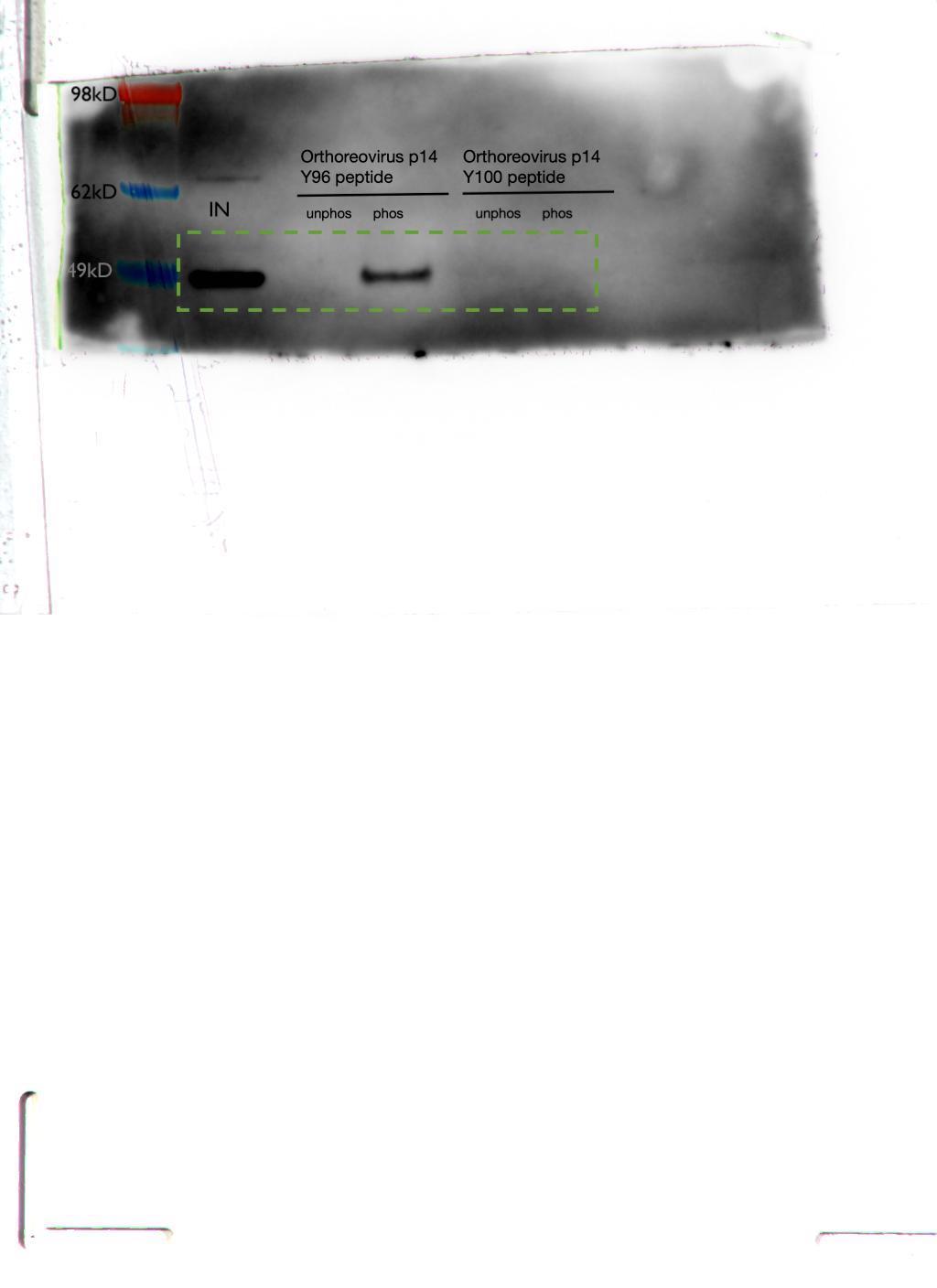

Supplement: Figure 3—figure supplement 1—source data 1. [file elife-74655-fig3-figsupp1-data1.zip › Figure 3 - supplement 1 - source data 1/Fig3-suppl1B blots/Fig3-suppl1B_Nck blot_annotated.jpg]

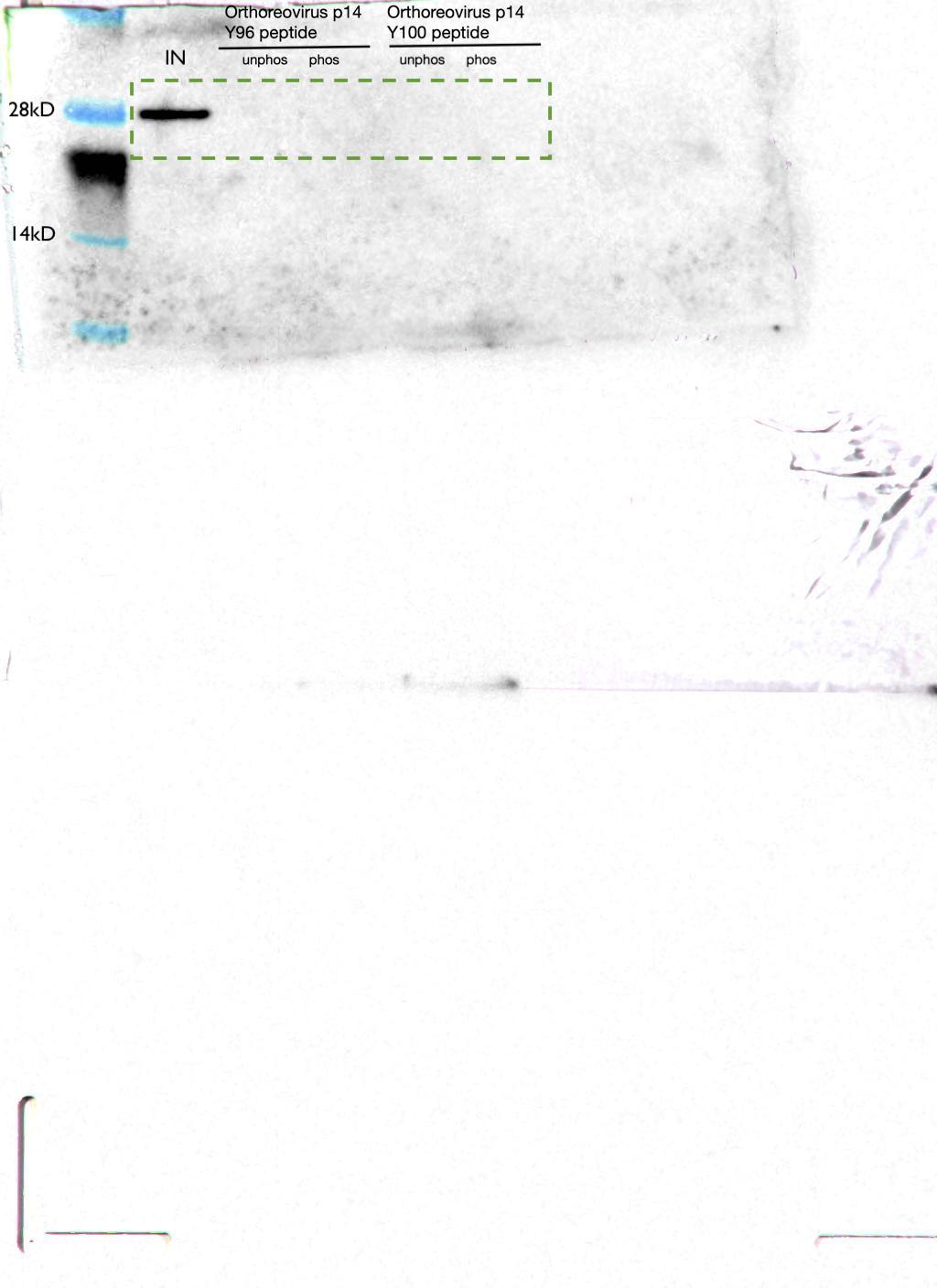

Supplement: Figure 3—figure supplement 1—source data 1. [file elife-74655-fig3-figsupp1-data1.zip › Figure 3 - supplement 1 - source data 1/Fig3-suppl1B blots/Fig3-suppl1B_Grb2 blot_annotated.jpg]

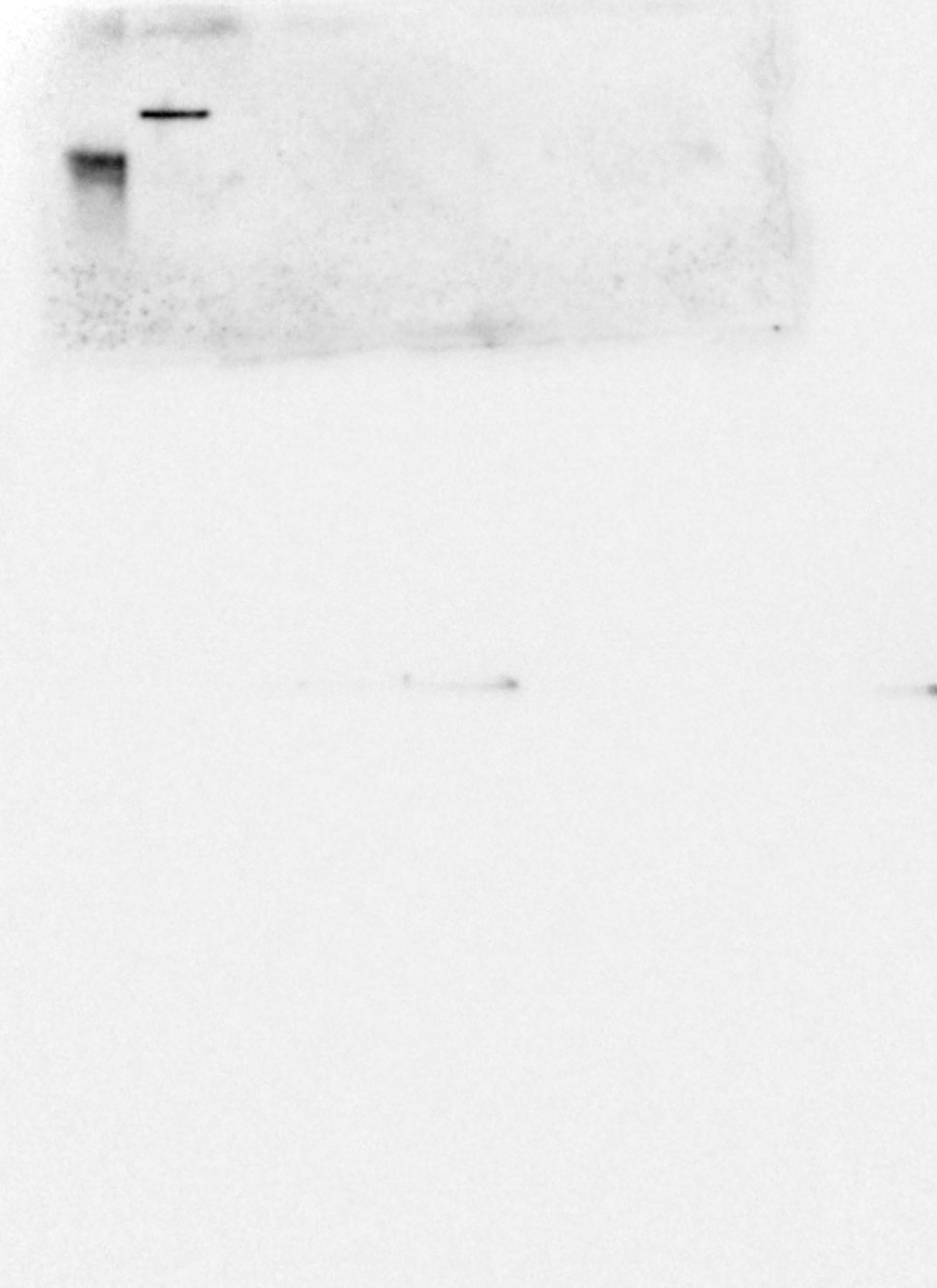

Supplement: Figure 3—figure supplement 1—source data 1. [file elife-74655-fig3-figsupp1-data1.zip › Figure 3 - supplement 1 - source data 1/Fig3-suppl1B blots/Fig3-suppl1B_Grb2 blot_raw.tif]

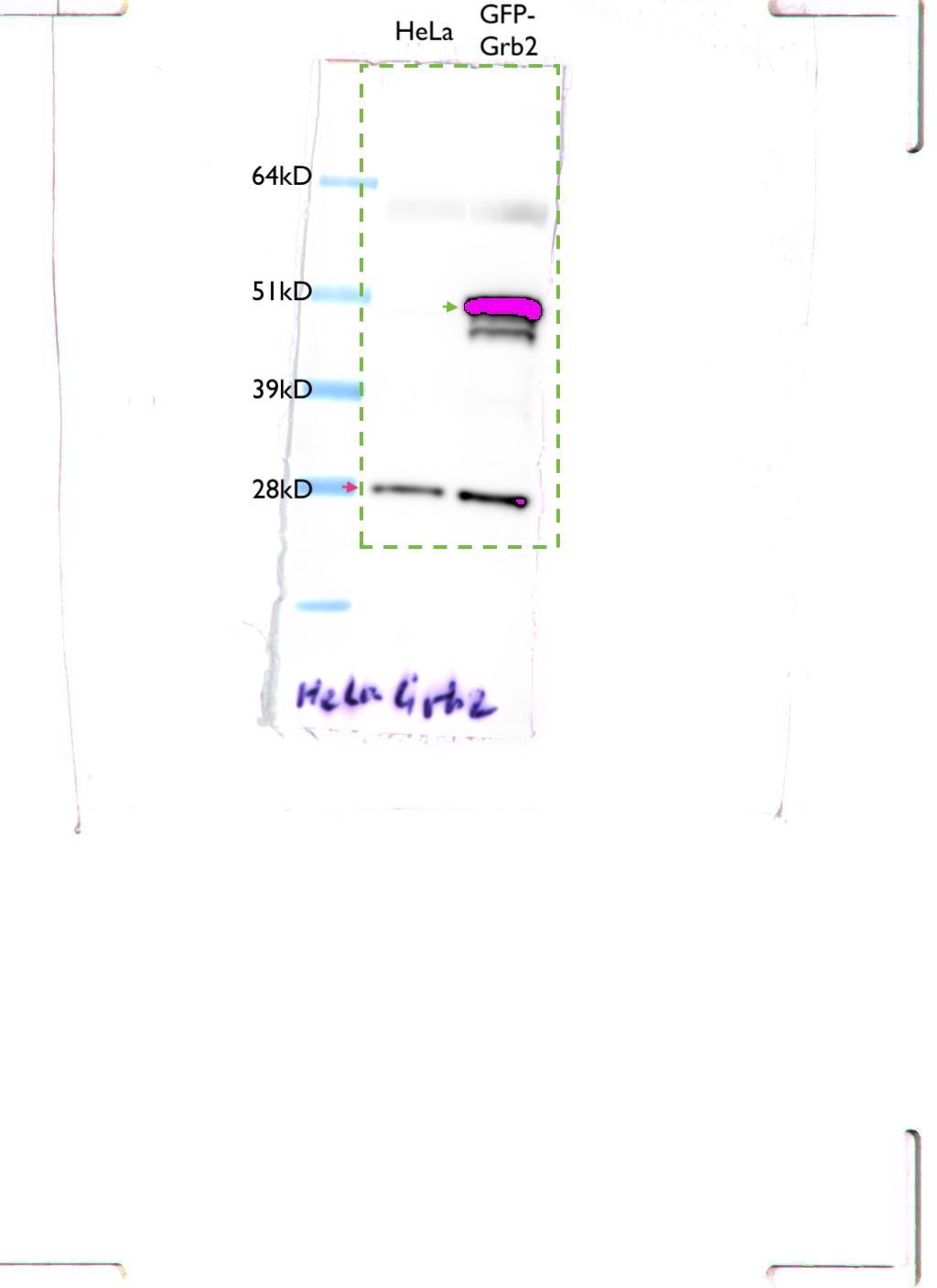

Supplement: Figure 4—figure supplement 1—source data 1. [file elife-74655-fig4-figsupp1-data1.zip › Figure 4 - supplement 1 - source data 1/Fig4-suppl1 blots/Fig4-suppl1_Grb2 blot_annotated.jpg]

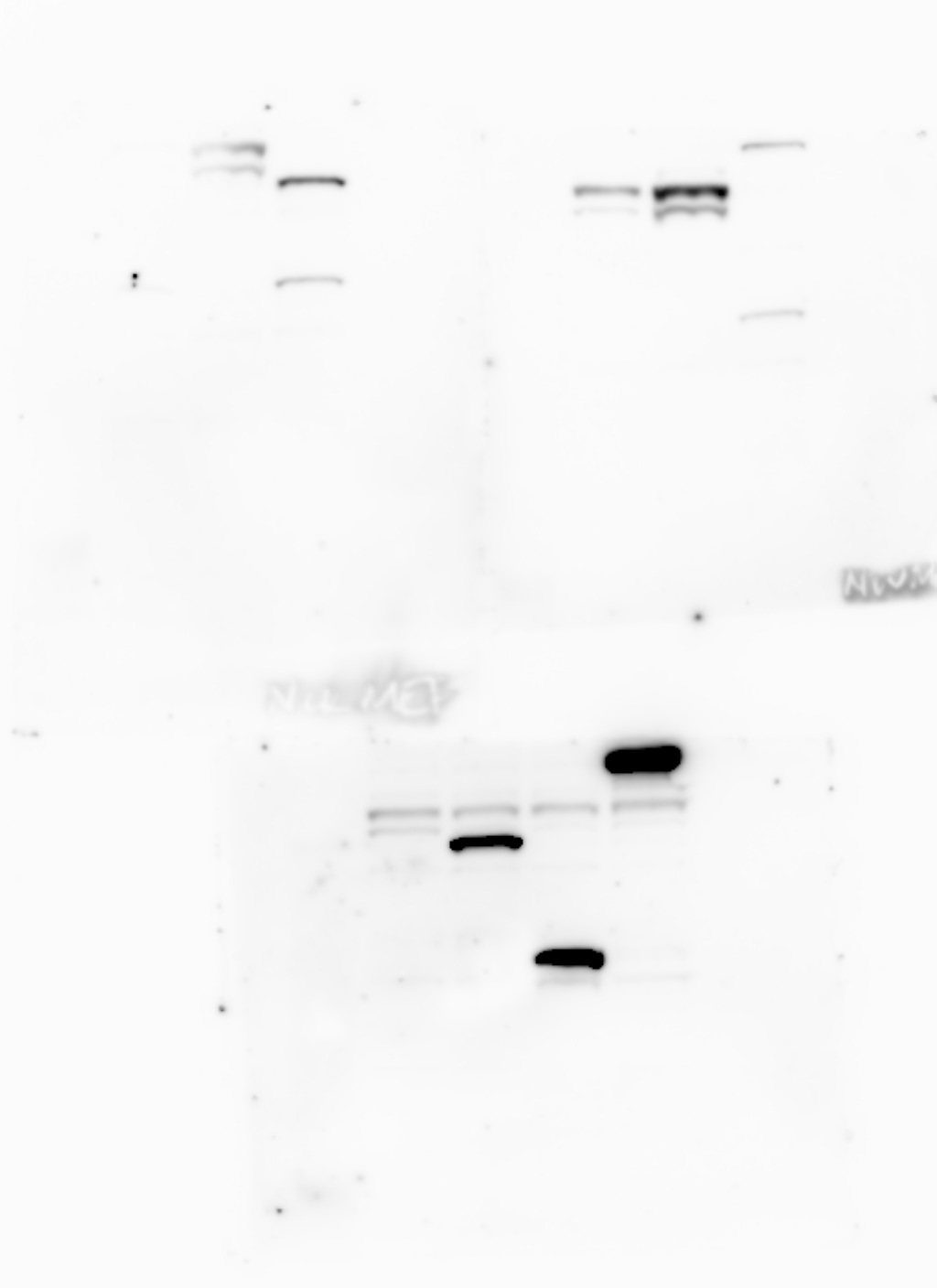

Supplement: Figure 4—figure supplement 1—source data 1. [file elife-74655-fig4-figsupp1-data1.zip › Figure 4 - supplement 1 - source data 1/Fig4-suppl1 blots/Fig4-suppl1_GFP blot_raw.tif]

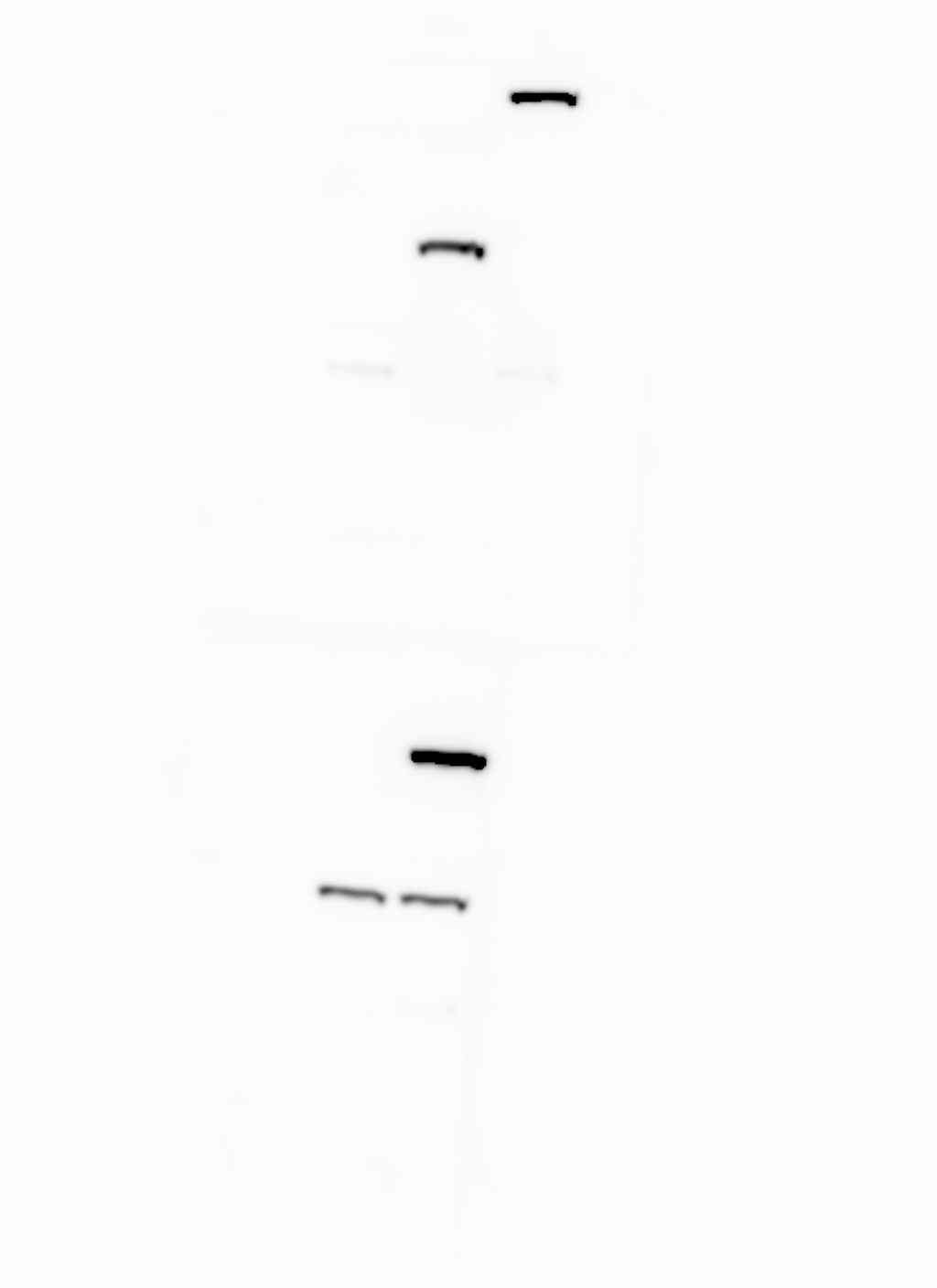

Supplement: Figure 4—figure supplement 1—source data 1. [file elife-74655-fig4-figsupp1-data1.zip › Figure 4 - supplement 1 - source data 1/Fig4-suppl1 blots/Fig4-suppl1_Nck blot_raw.tif]

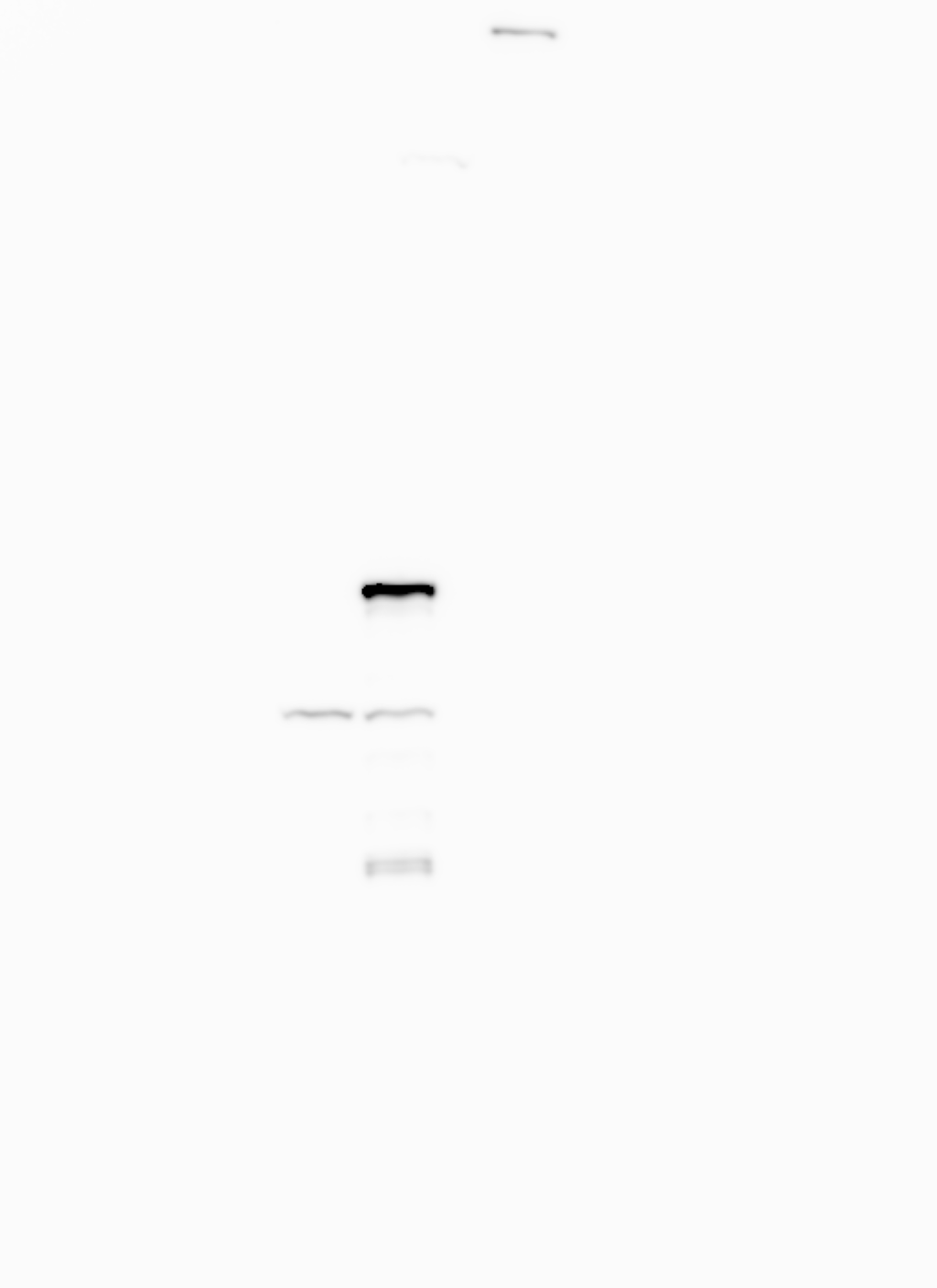

Supplement: Figure 4—figure supplement 1—source data 1. [file elife-74655-fig4-figsupp1-data1.zip › Figure 4 - supplement 1 - source data 1/Fig4-suppl1 blots/Fig4-suppl1_N-WASP blot_raw.tif]

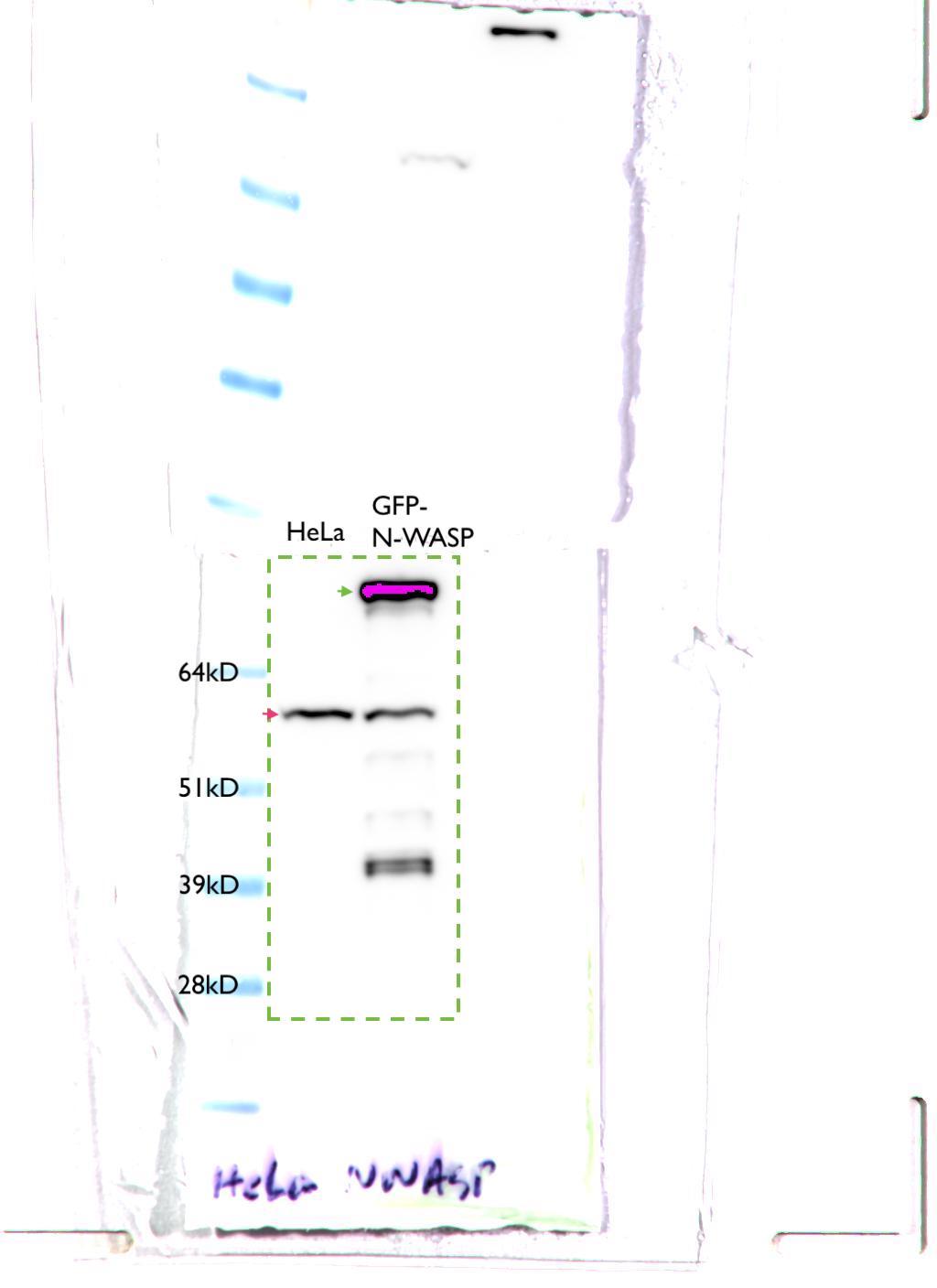

Supplement: Figure 4—figure supplement 1—source data 1. [file elife-74655-fig4-figsupp1-data1.zip › Figure 4 - supplement 1 - source data 1/Fig4-suppl1 blots/Fig4-suppl1_N-WASP blot_annotated.jpg]

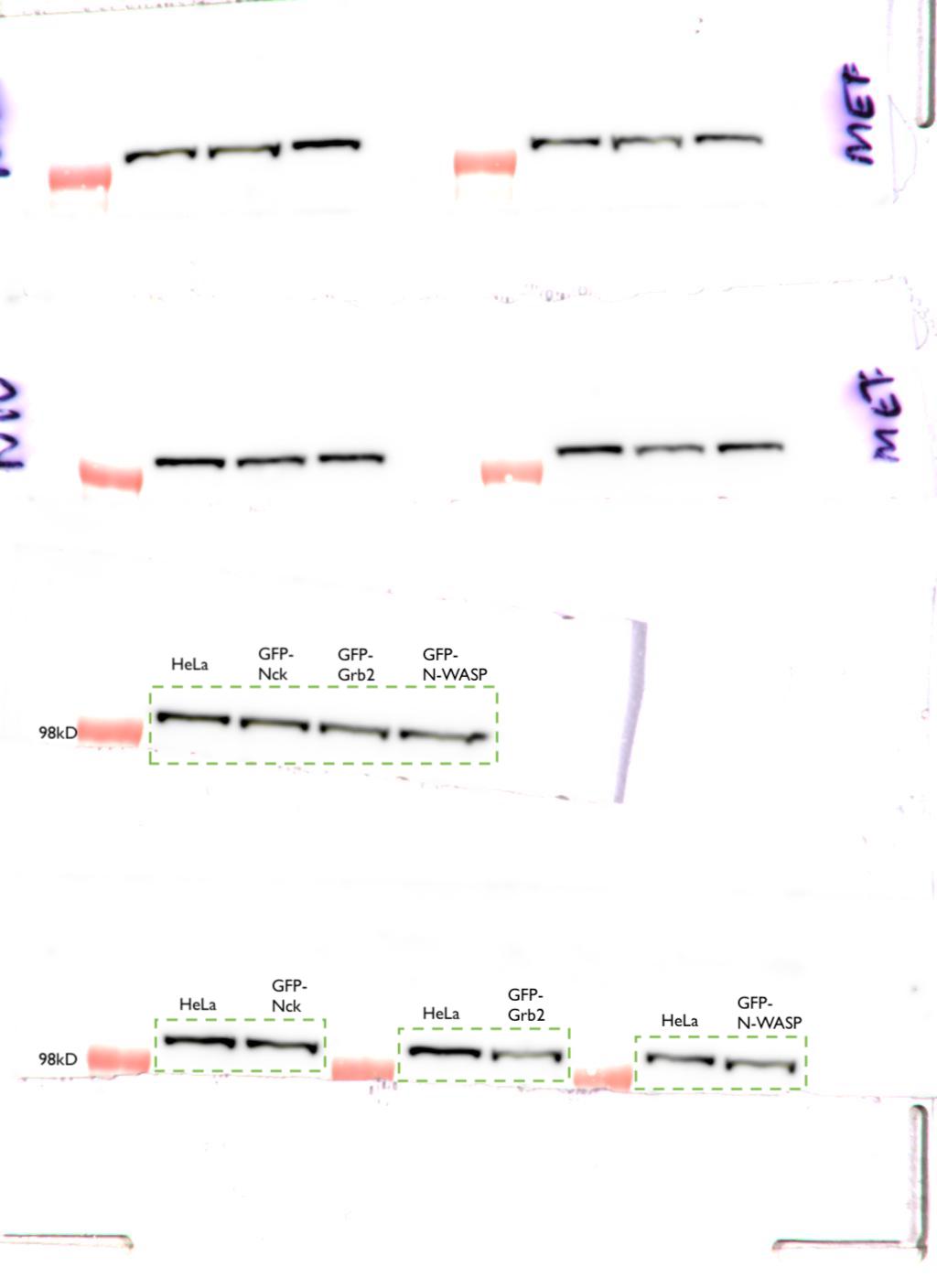

Supplement: Figure 4—figure supplement 1—source data 1. [file elife-74655-fig4-figsupp1-data1.zip › Figure 4 - supplement 1 - source data 1/Fig4-suppl1 blots/Fig4-suppl1_vinculin blots_annotated.jpg]

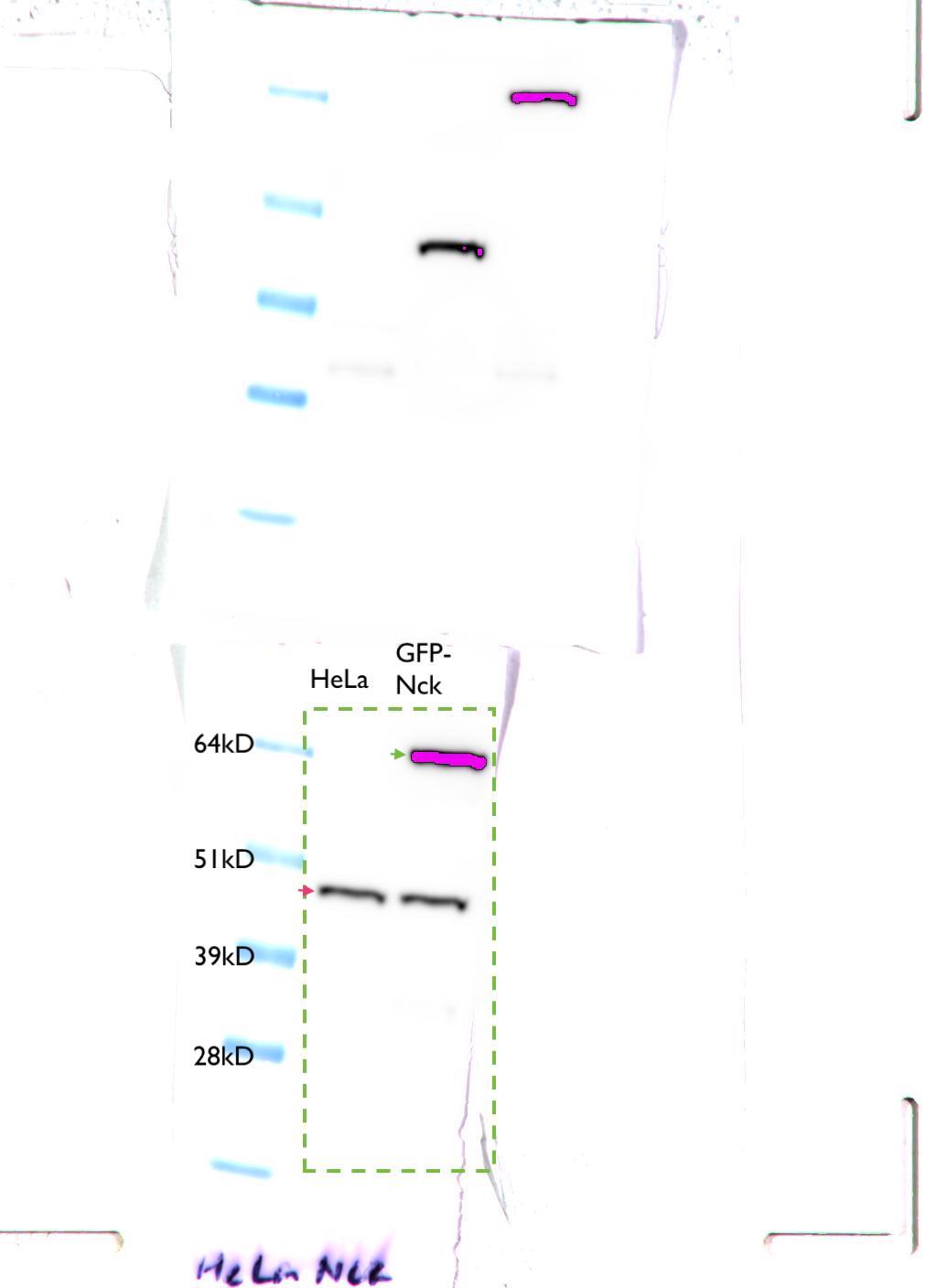

Supplement: Figure 4—figure supplement 1—source data 1. [file elife-74655-fig4-figsupp1-data1.zip › Figure 4 - supplement 1 - source data 1/Fig4-suppl1 blots/Fig4-suppl1_Nck blot_annotated.jpg]

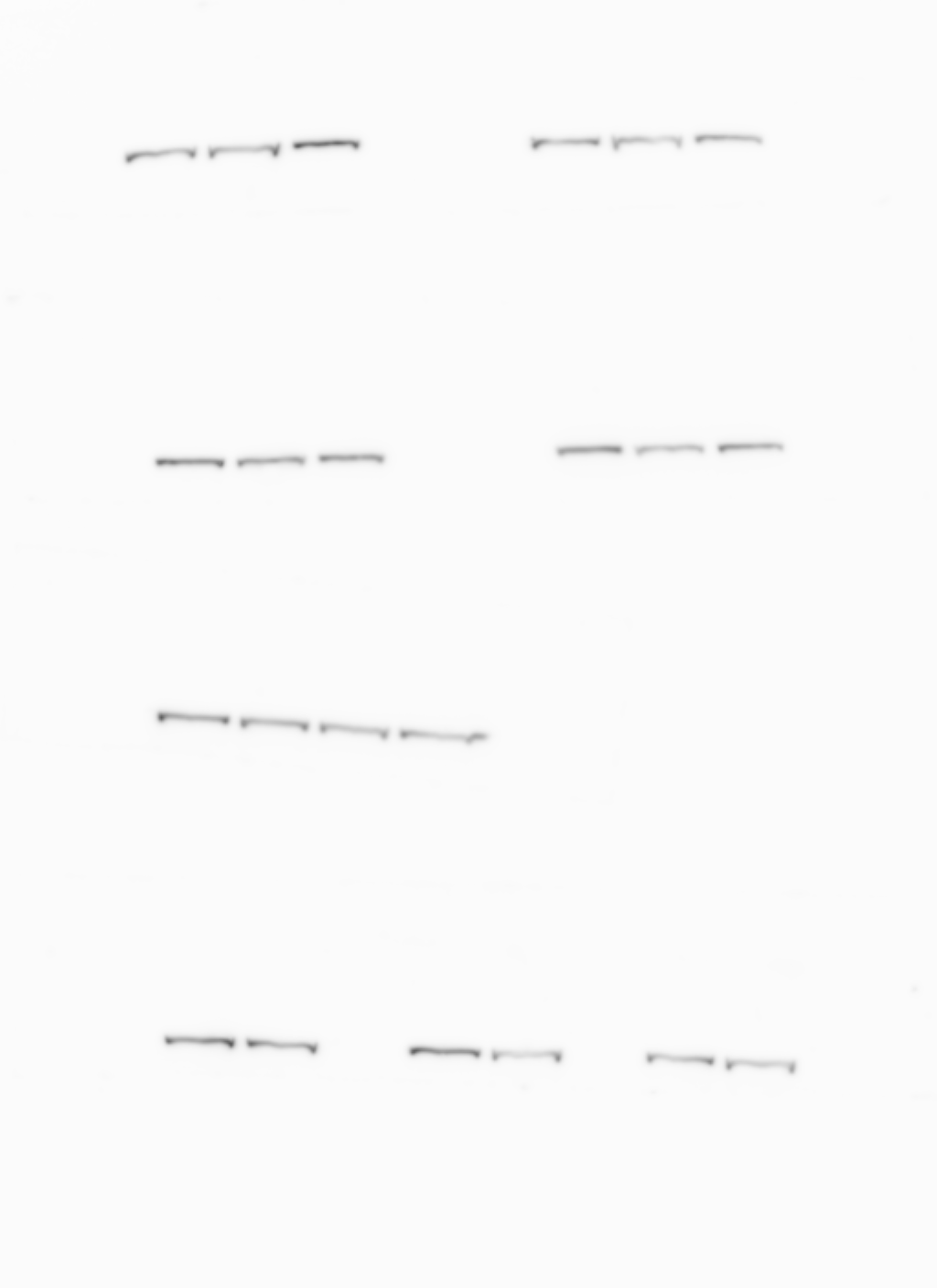

Supplement: Figure 4—figure supplement 1—source data 1. [file elife-74655-fig4-figsupp1-data1.zip › Figure 4 - supplement 1 - source data 1/Fig4-suppl1 blots/Fig4-suppl1_vinculin blots_raw.tif]

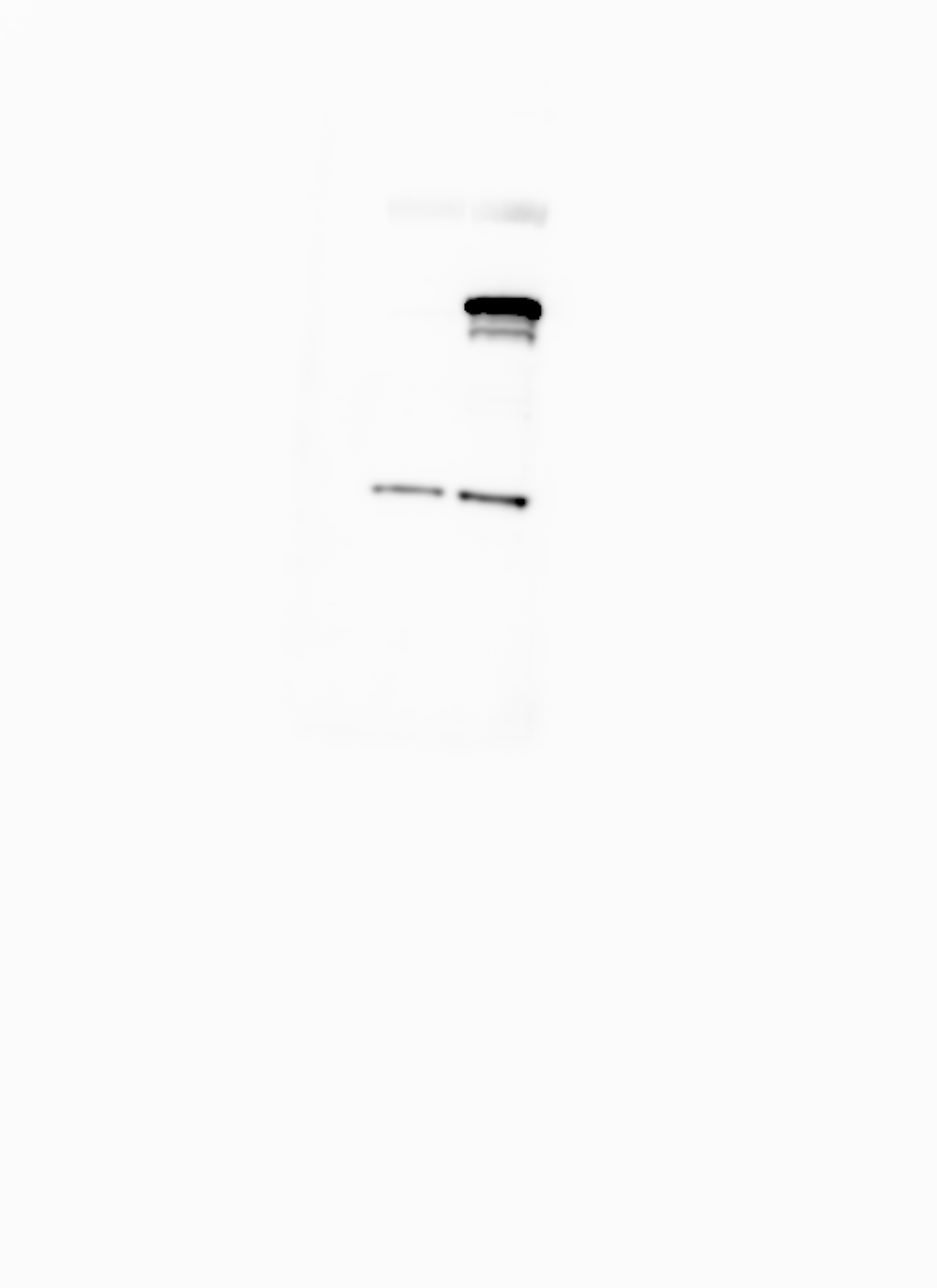

Supplement: Figure 4—figure supplement 1—source data 1. [file elife-74655-fig4-figsupp1-data1.zip › Figure 4 - supplement 1 - source data 1/Fig4-suppl1 blots/Fig4-suppl1_Grb2 blot_raw.tif]

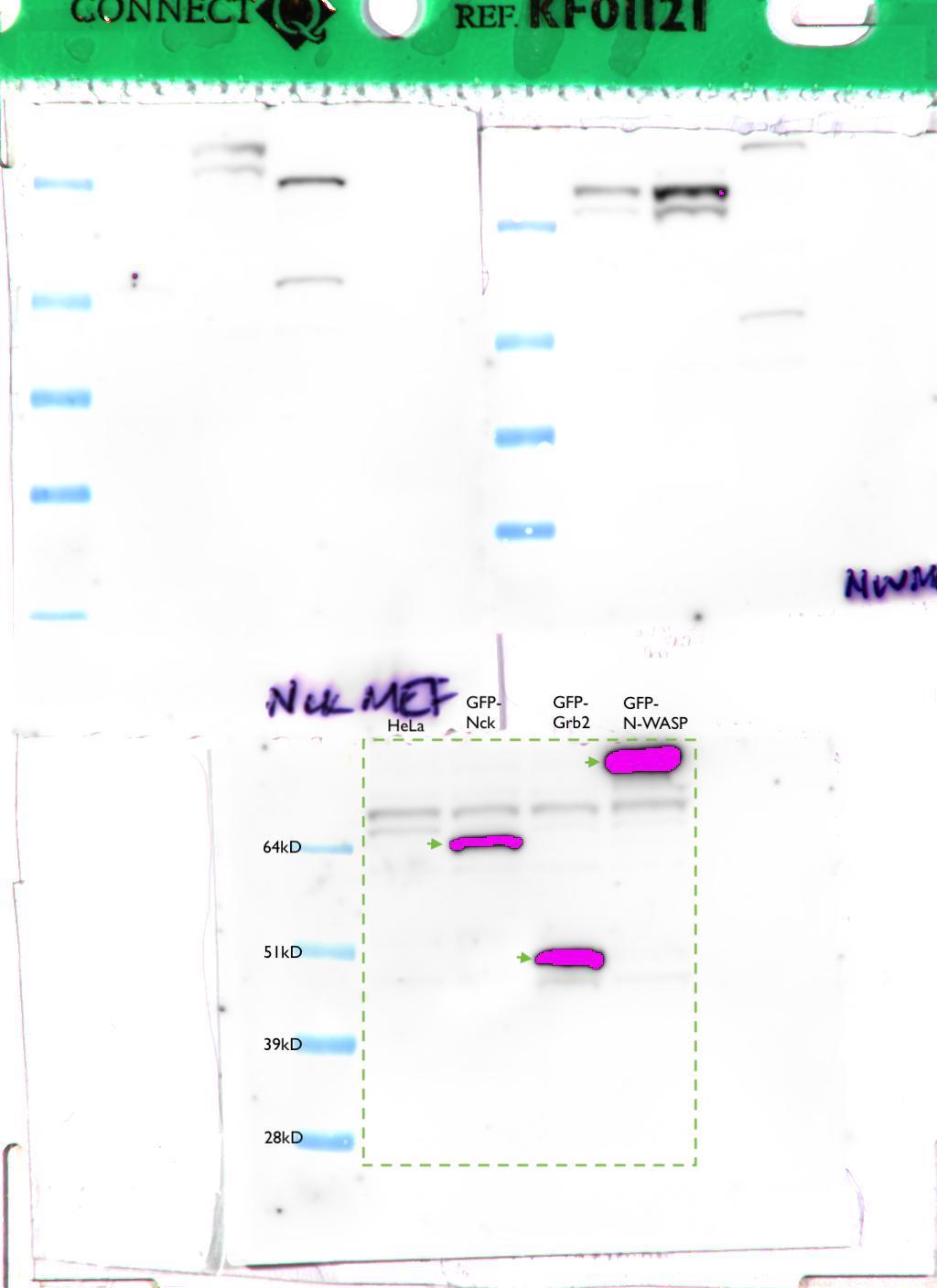

Supplement: Figure 4—figure supplement 1—source data 1. [file elife-74655-fig4-figsupp1-data1.zip › Figure 4 - supplement 1 - source data 1/Fig4-suppl1 blots/Fig4-suppl1_GFP blot_annotated.jpg]

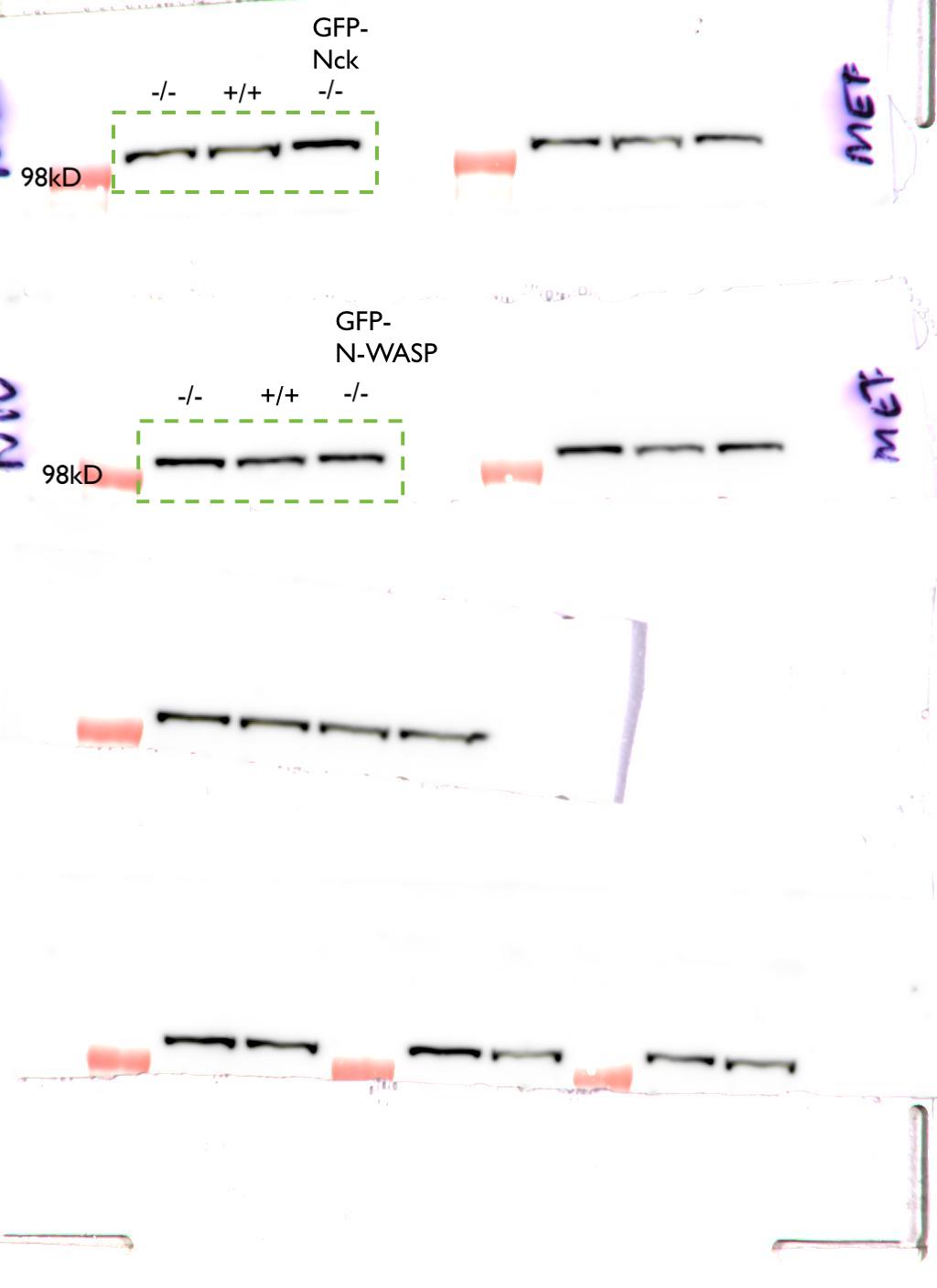

Supplement: Figure 5—figure supplement 2—source data 1. [file elife-74655-fig5-figsupp2-data1.zip › Figure 5 - supplement 2 - source data 1/Fig5-suppl2 blots/Fig5-suppl2_vinculin blots_annotated.jpg]

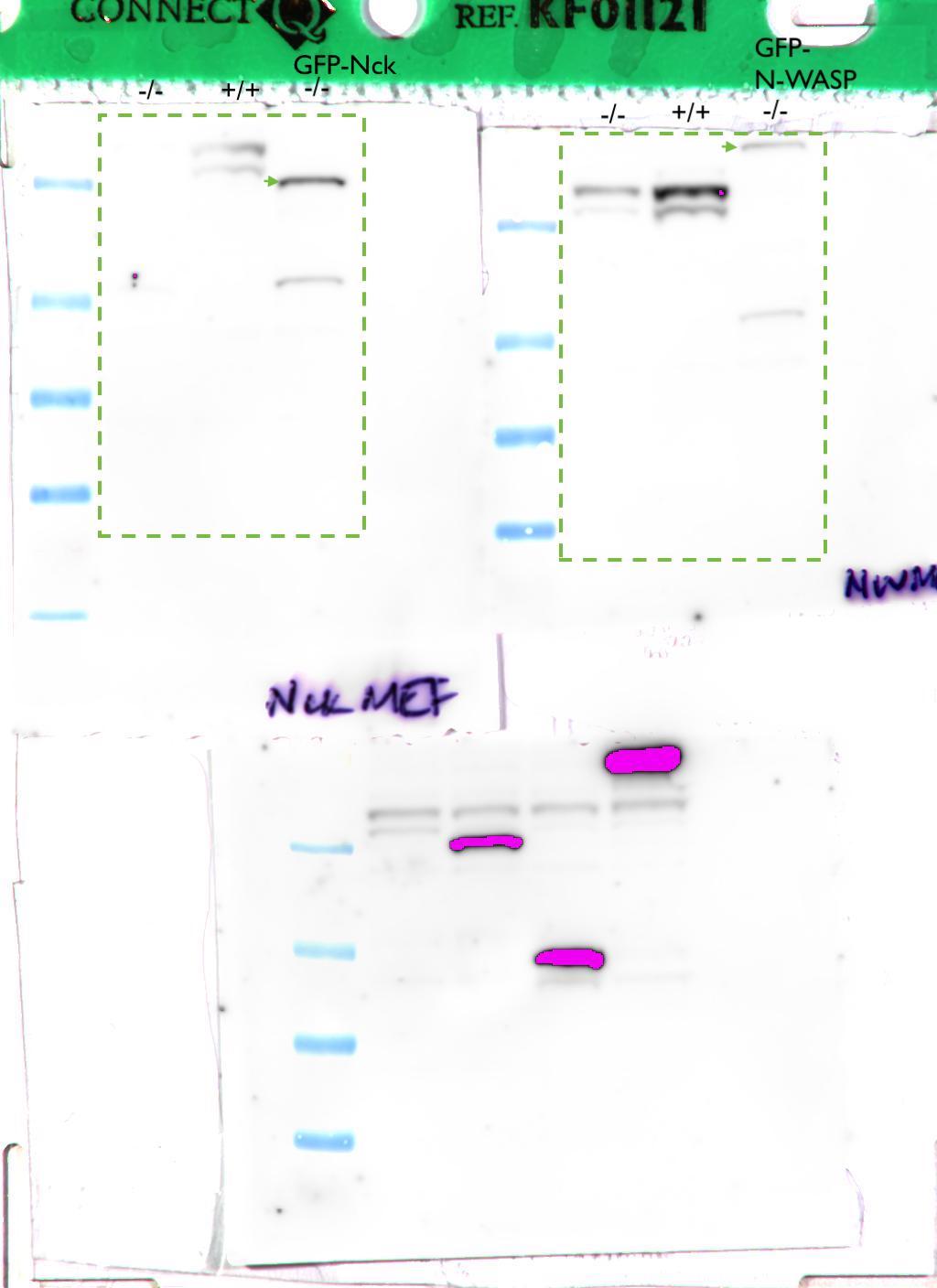

Supplement: Figure 5—figure supplement 2—source data 1. [file elife-74655-fig5-figsupp2-data1.zip › Figure 5 - supplement 2 - source data 1/Fig5-suppl2 blots/Fig5-suppl2_GFP blots_annotated.jpg]

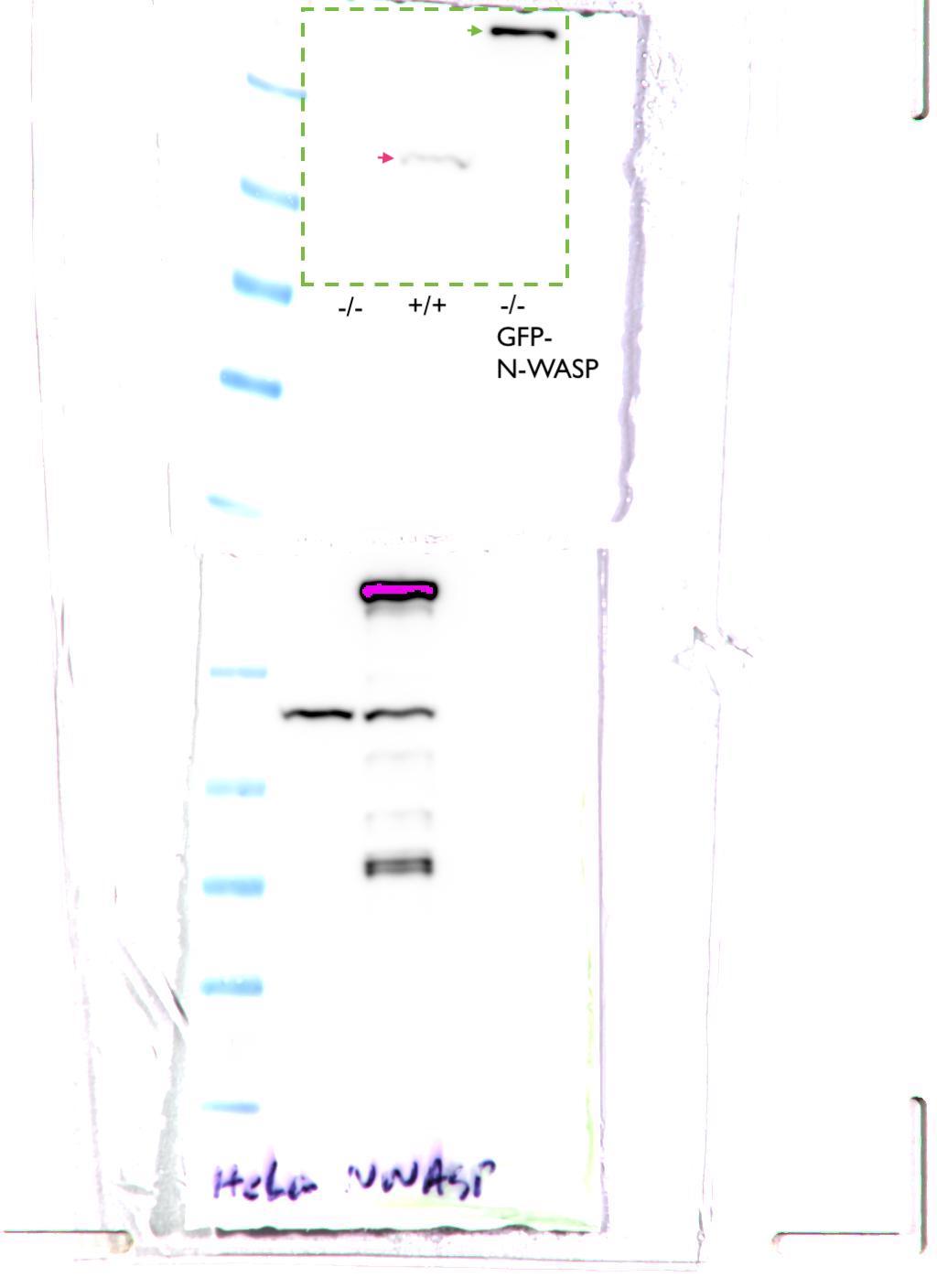

Supplement: Figure 5—figure supplement 2—source data 1. [file elife-74655-fig5-figsupp2-data1.zip › Figure 5 - supplement 2 - source data 1/Fig5-suppl2 blots/Fig5-suppl2_N-WASP blot_annotated.jpg]

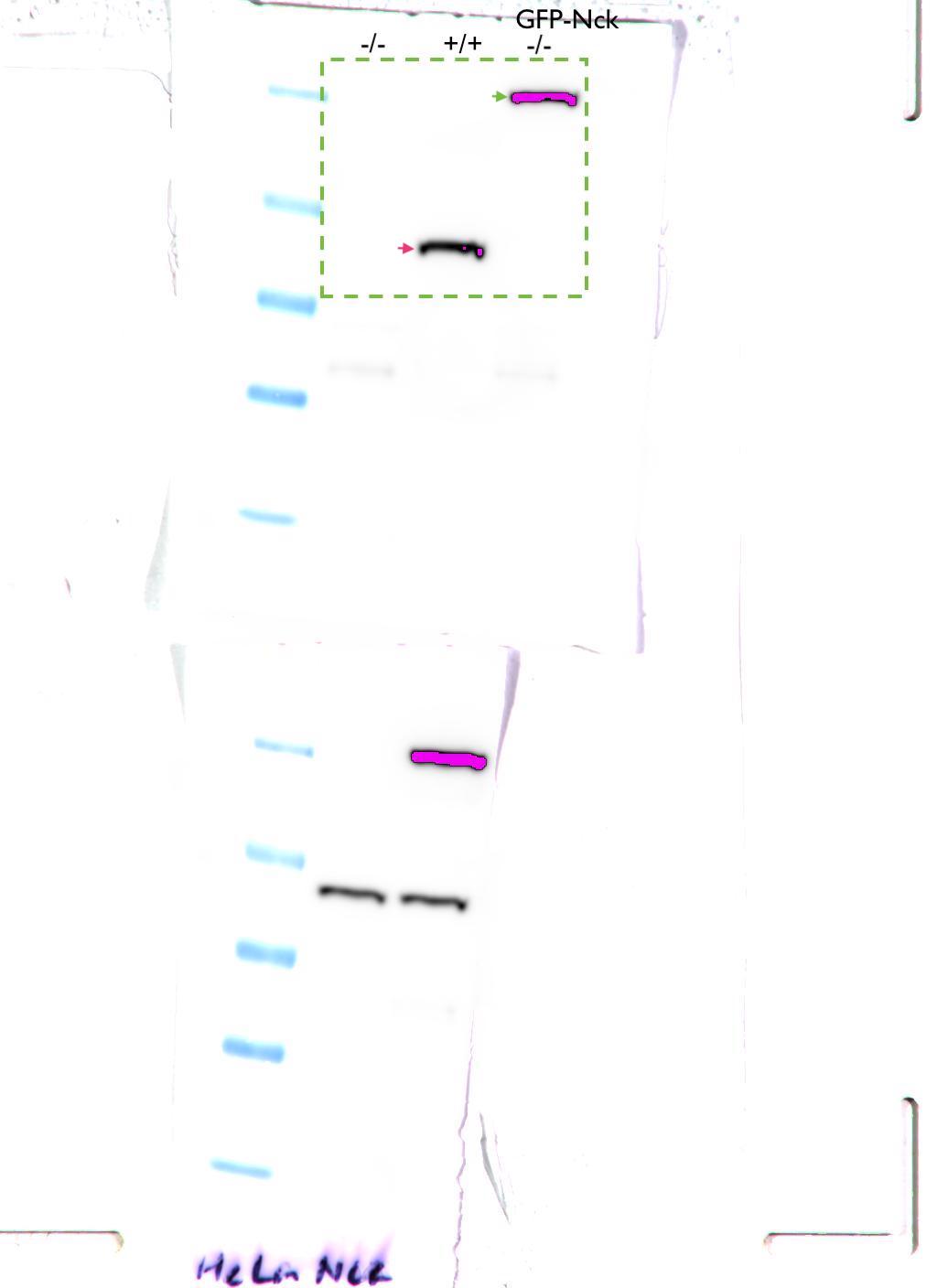

Supplement: Figure 5—figure supplement 2—source data 1. [file elife-74655-fig5-figsupp2-data1.zip › Figure 5 - supplement 2 - source data 1/Fig5-suppl2 blots/Fig5-suppl2_Nck blot_annotated.jpg]

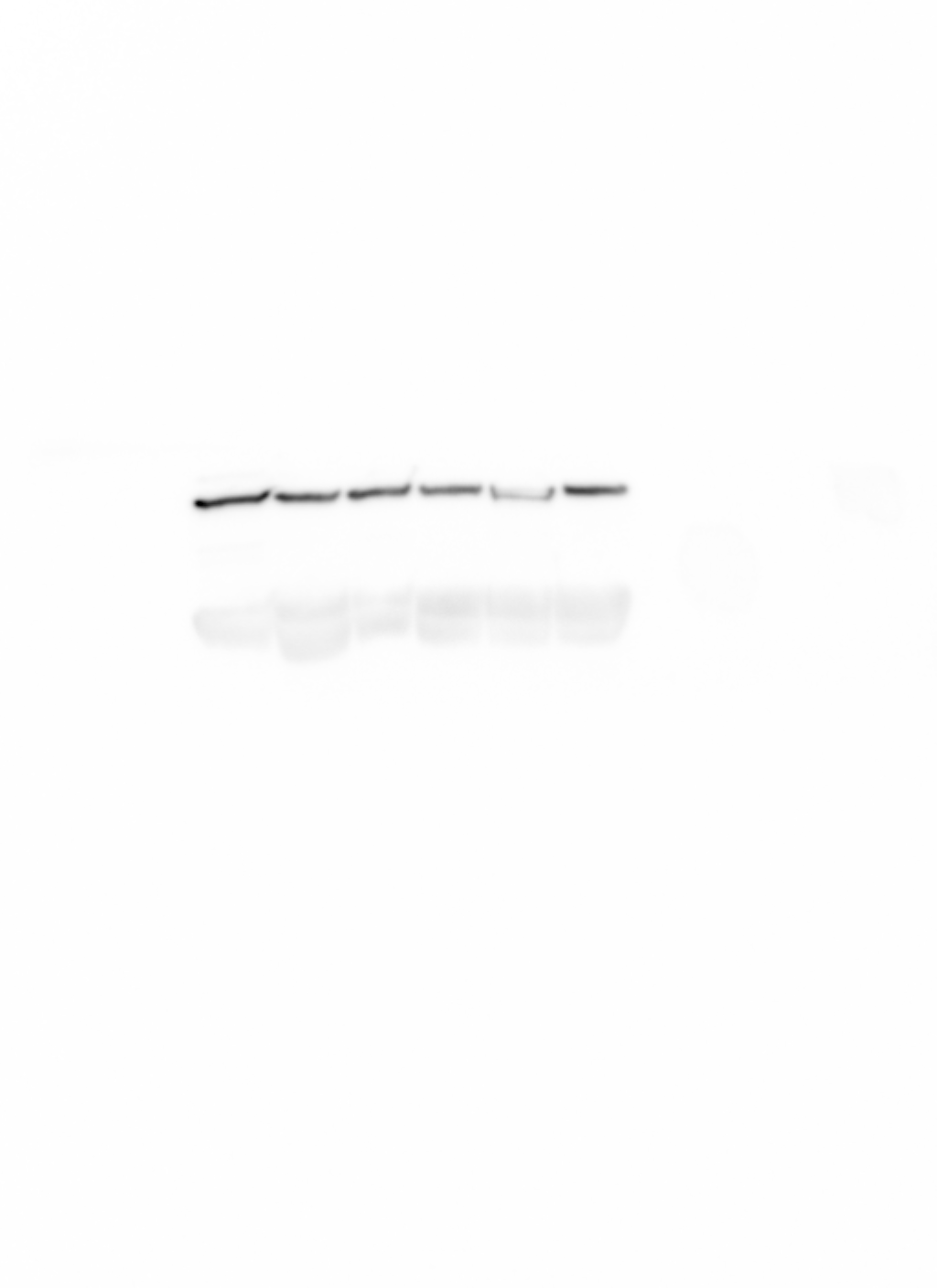

Supplement: Figure 6—figure supplement 1—source data 1. [file elife-74655-fig6-figsupp1-data1.zip › Figure 6 - supplement 1 - source data 1/Fig6-suppl1C blots/Fig6-suppl1C_VINC blot_raw.tif]

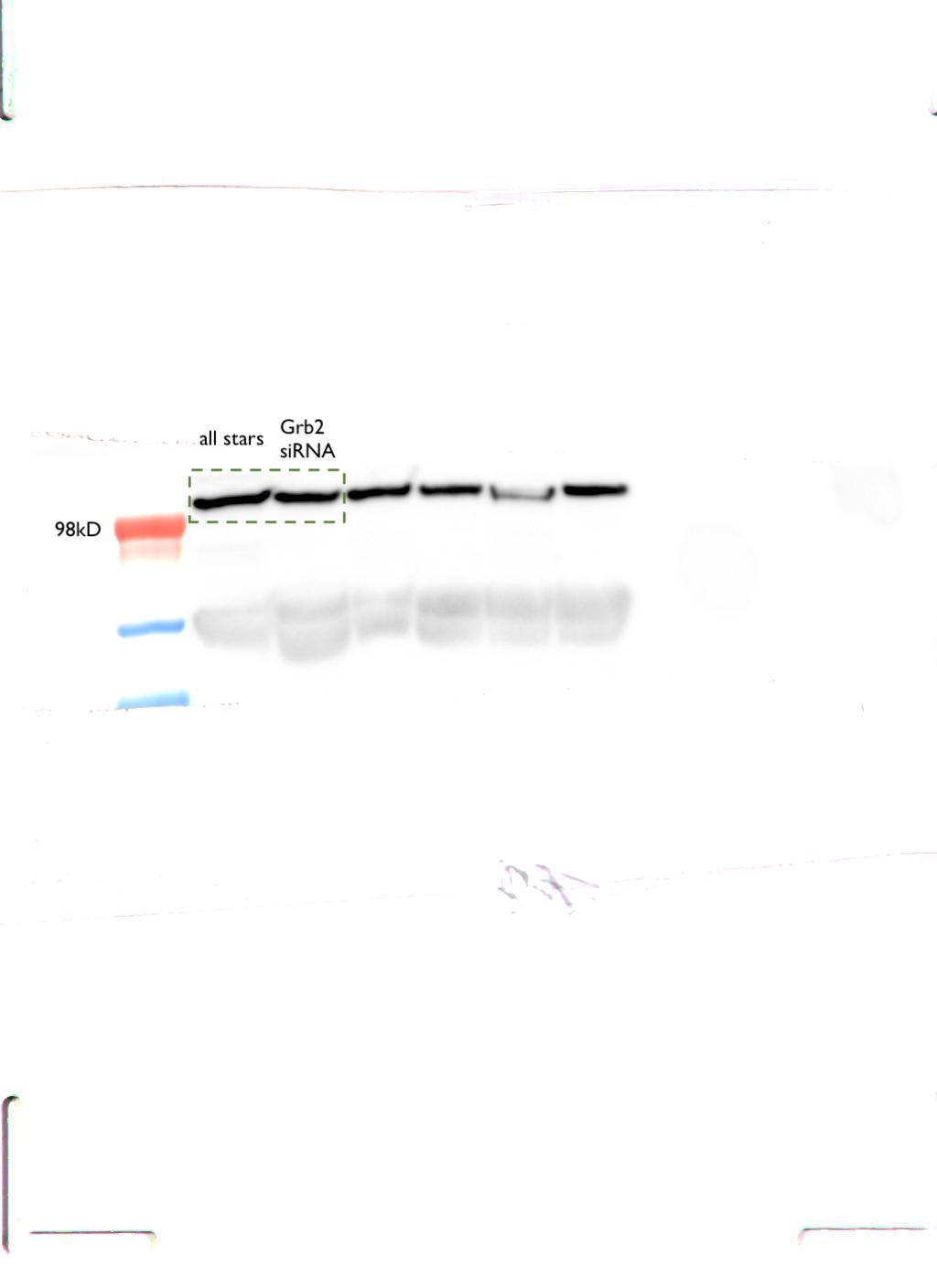

Supplement: Figure 6—figure supplement 1—source data 1. [file elife-74655-fig6-figsupp1-data1.zip › Figure 6 - supplement 1 - source data 1/Fig6-suppl1C blots/Fig6-suppl1C_VINC blot_annotated.jpg]

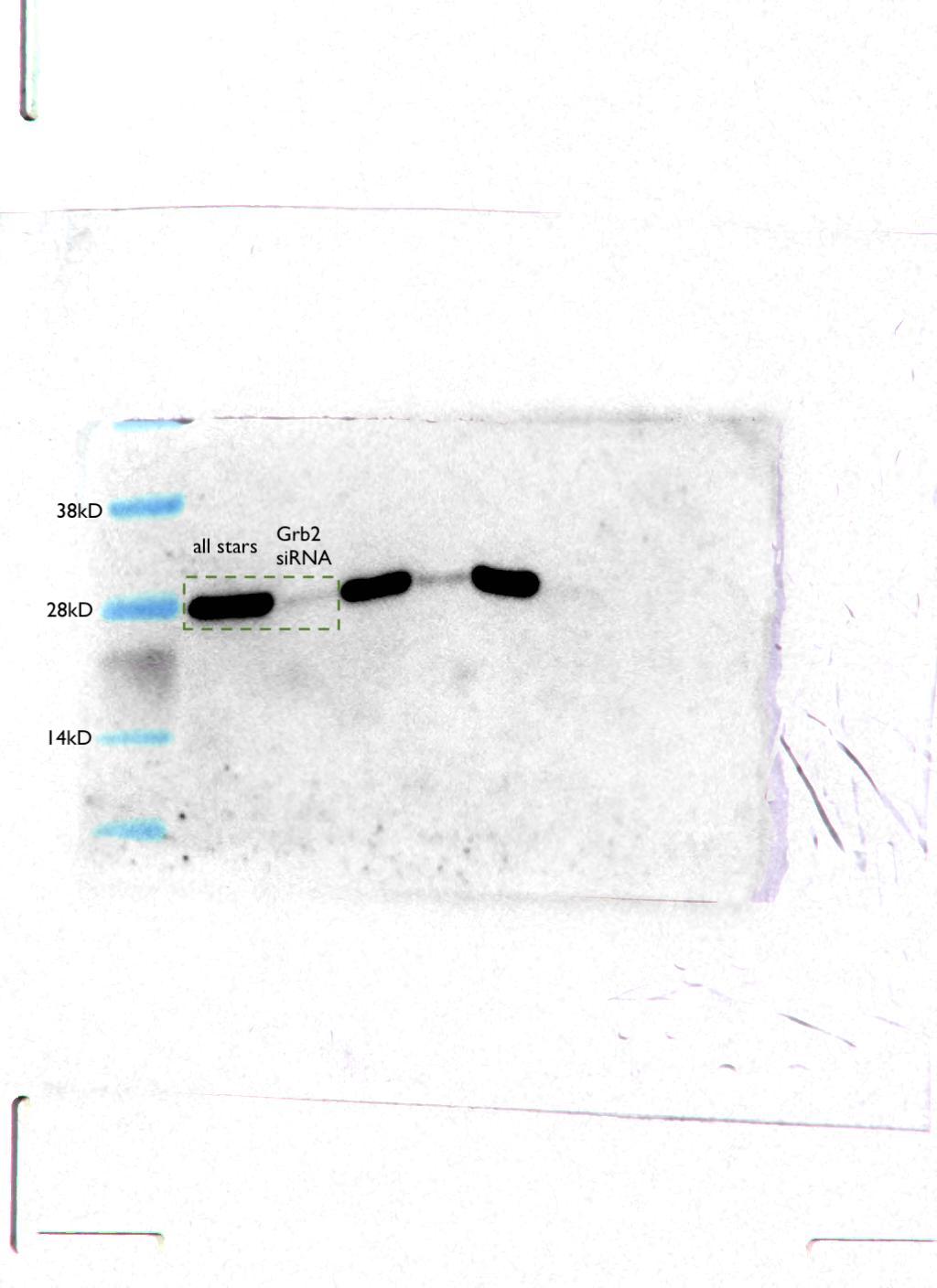

Supplement: Figure 6—figure supplement 1—source data 1. [file elife-74655-fig6-figsupp1-data1.zip › Figure 6 - supplement 1 - source data 1/Fig6-suppl1C blots/Fig6-suppl1C_Grb2 blot_annotated.jpg]

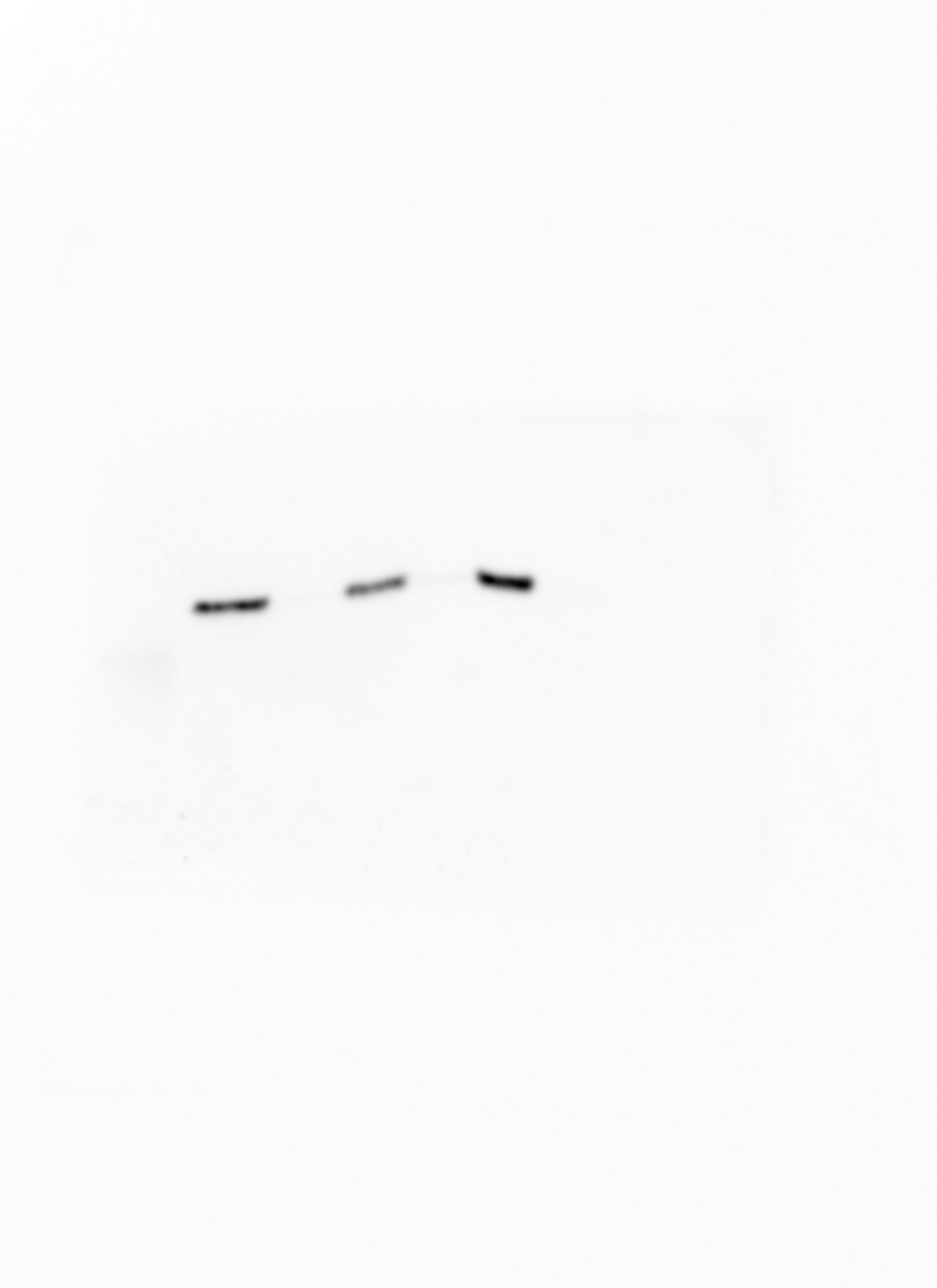

Supplement: Figure 6—figure supplement 1—source data 1. [file elife-74655-fig6-figsupp1-data1.zip › Figure 6 - supplement 1 - source data 1/Fig6-suppl1C blots/Fig6-suppl1C_Grb2 blot_raw.tif]

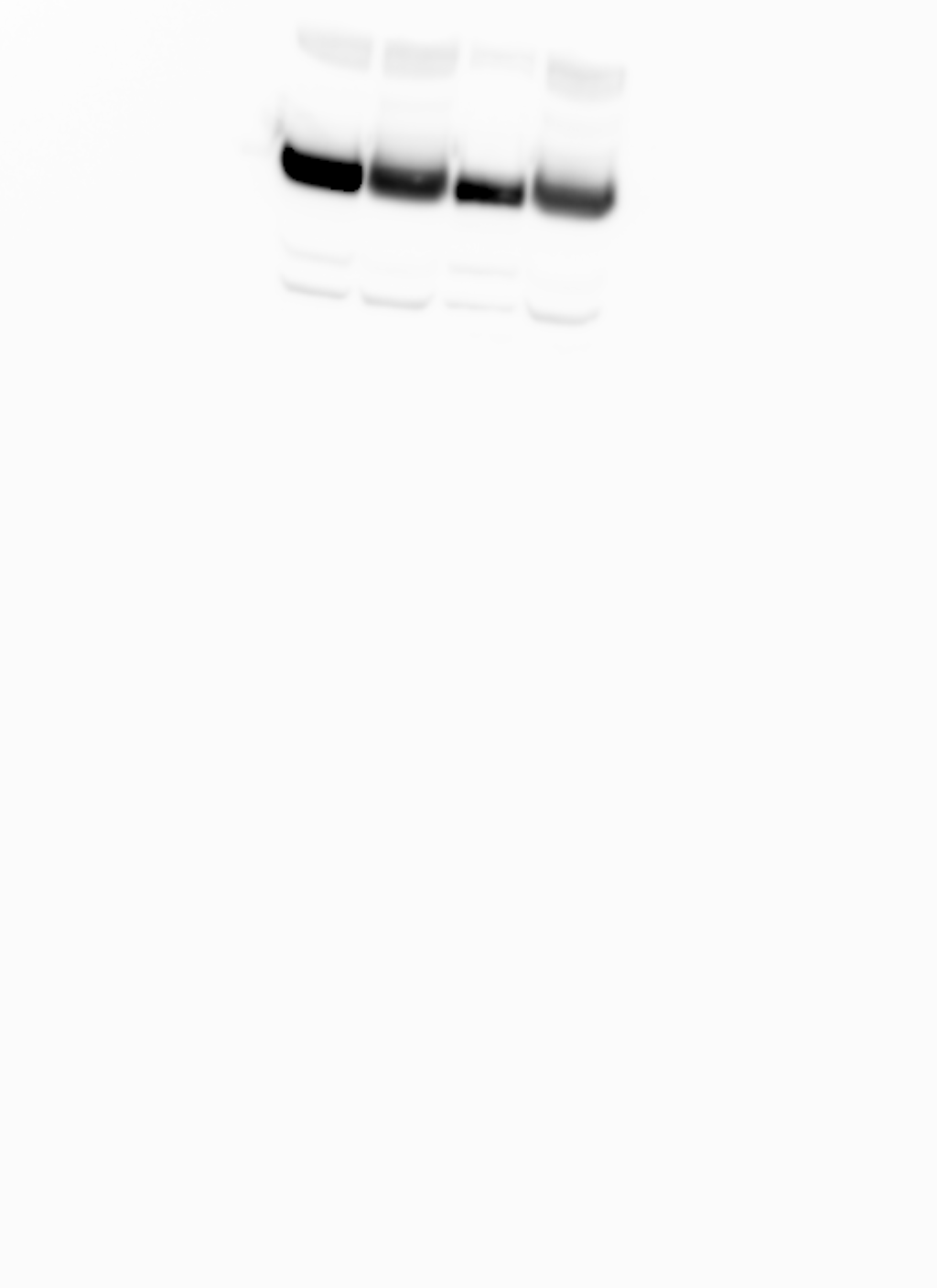

Supplement: Figure 6—figure supplement 1—source data 1. [file elife-74655-fig6-figsupp1-data1.zip › Figure 6 - supplement 1 - source data 1/Fig6-suppl1A blots/Fig6-suppl1A_TagGFP2 blot_raw.tif]

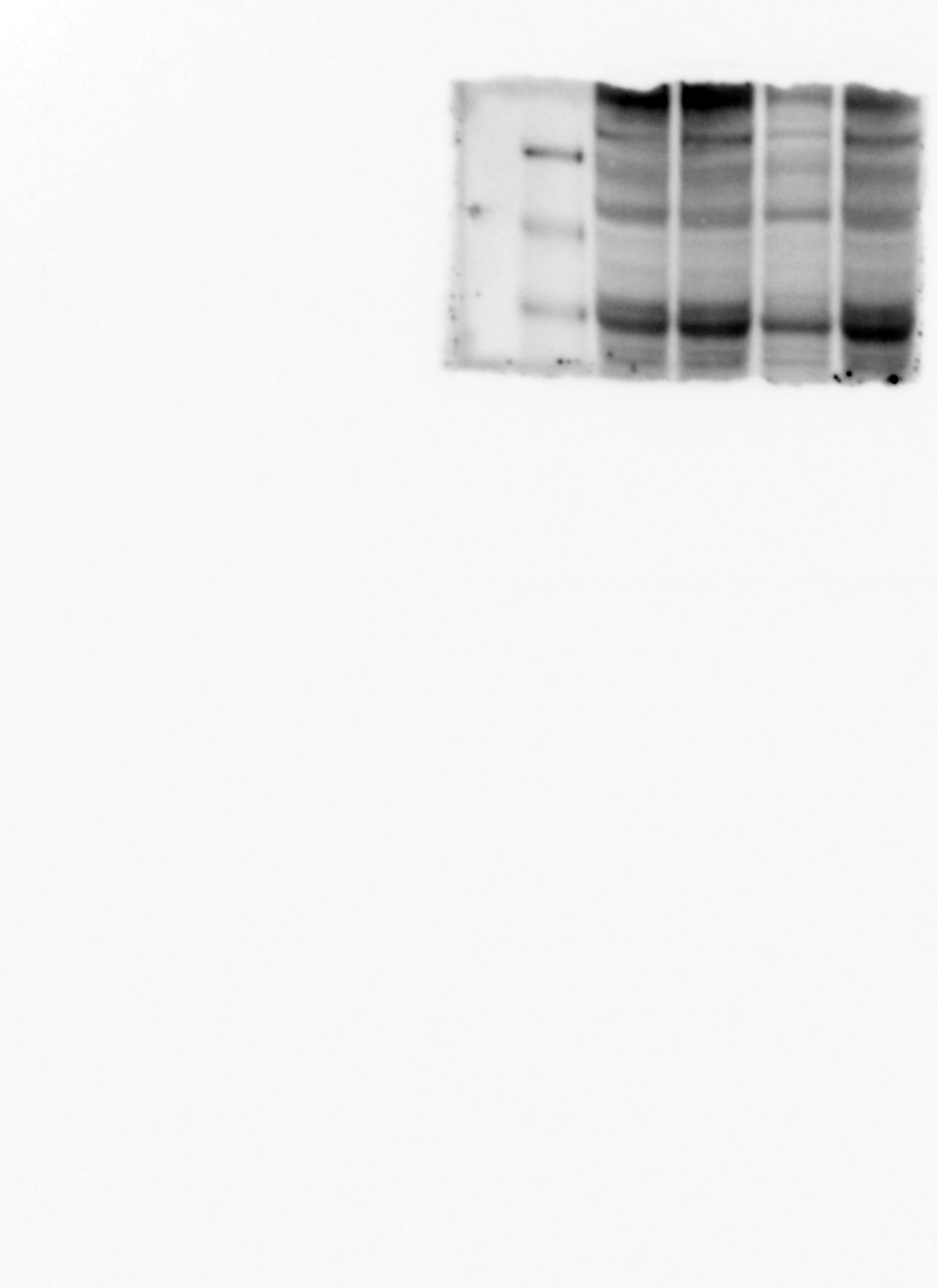

Supplement: Figure 6—figure supplement 1—source data 1. [file elife-74655-fig6-figsupp1-data1.zip › Figure 6 - supplement 1 - source data 1/Fig6-suppl1A blots/Fig6-suppl1A_PY99 blot_raw.tif]

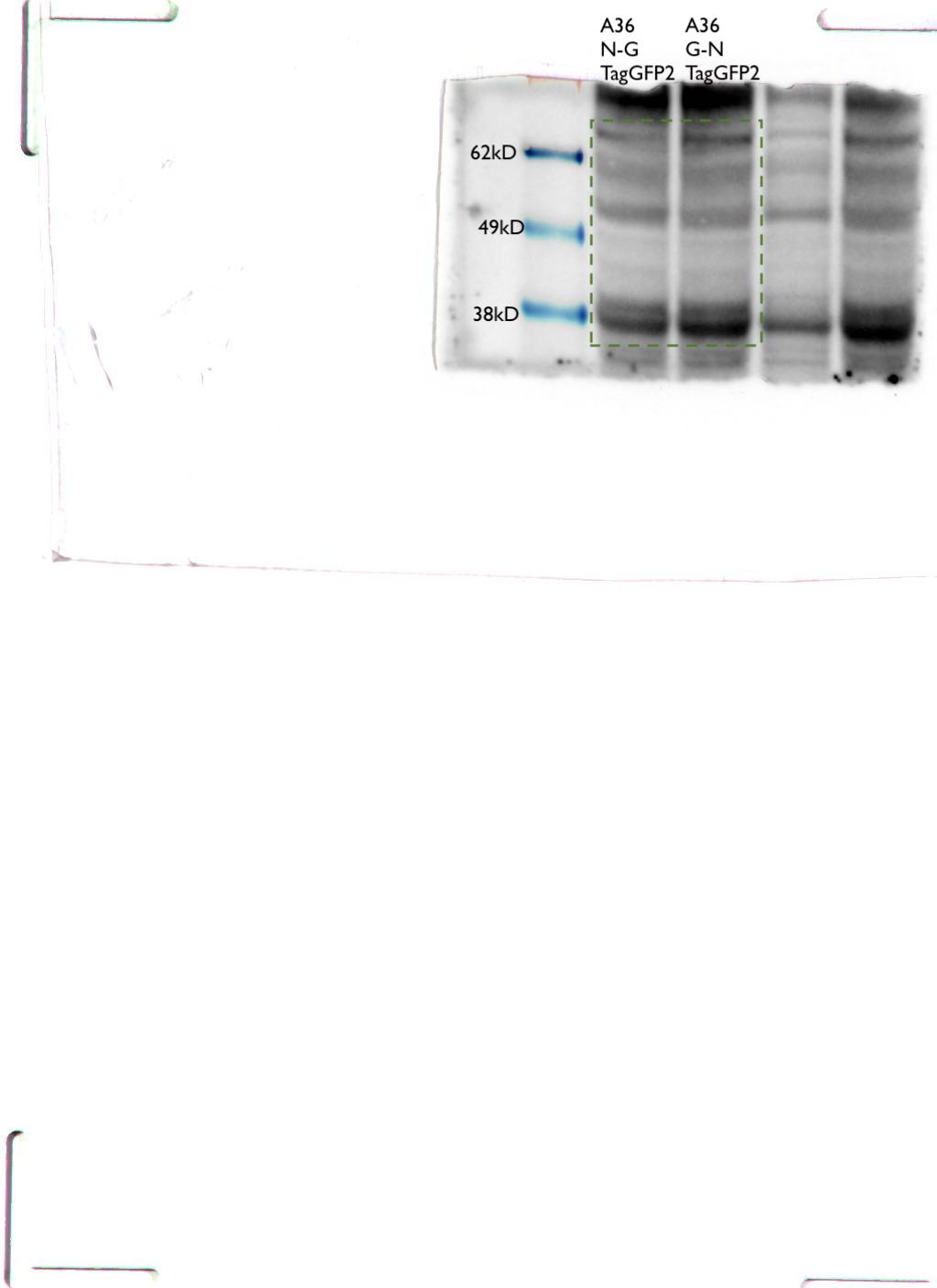

Supplement: Figure 6—figure supplement 1—source data 1. [file elife-74655-fig6-figsupp1-data1.zip › Figure 6 - supplement 1 - source data 1/Fig6-suppl1A blots/Fig6-suppl1A_PY99 blot_annotated.jpg]

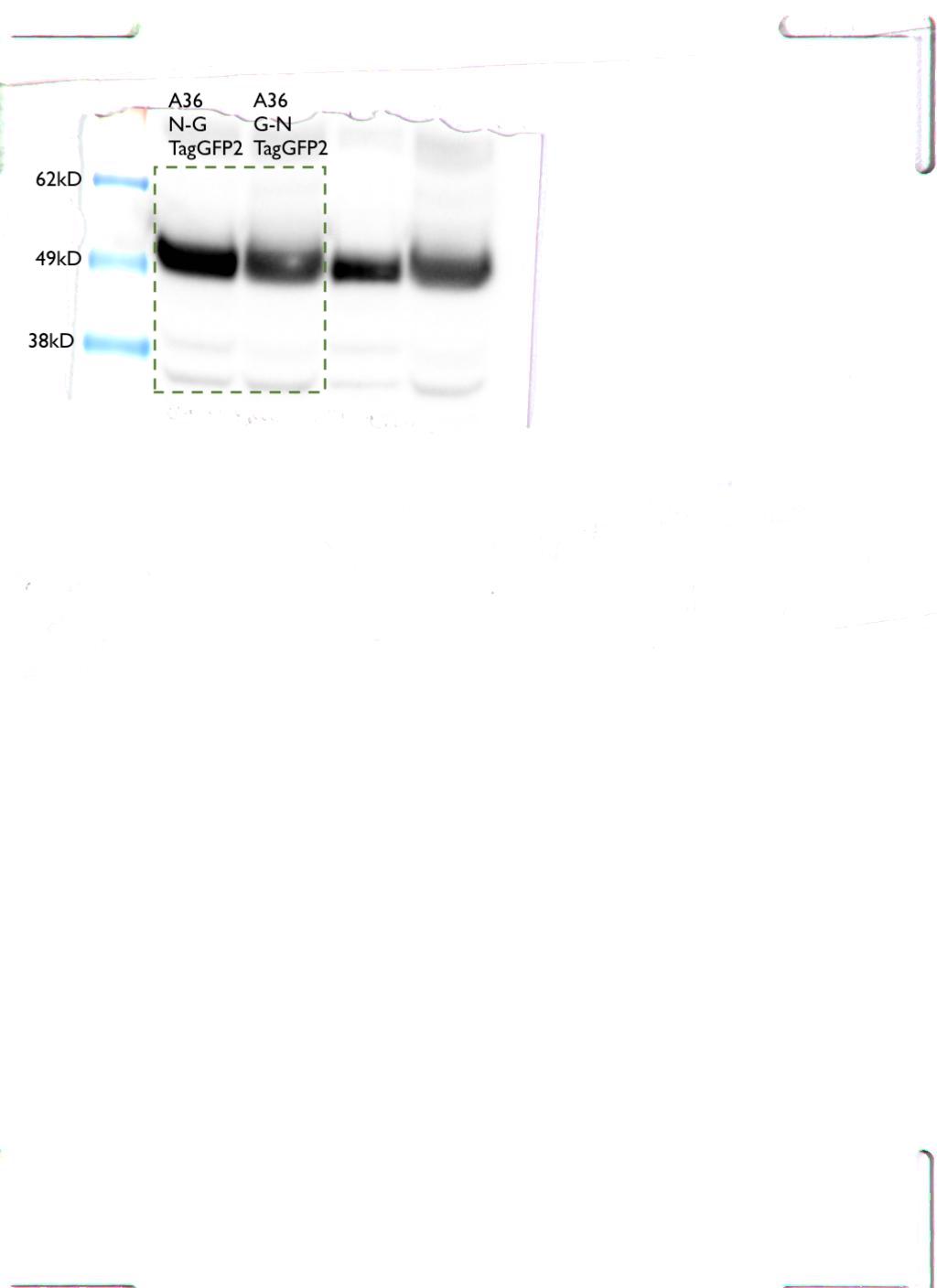

Supplement: Figure 6—figure supplement 1—source data 1. [file elife-74655-fig6-figsupp1-data1.zip › Figure 6 - supplement 1 - source data 1/Fig6-suppl1A blots/Fig6-suppl1A_TagGFP2 blot_annotated.jpg]

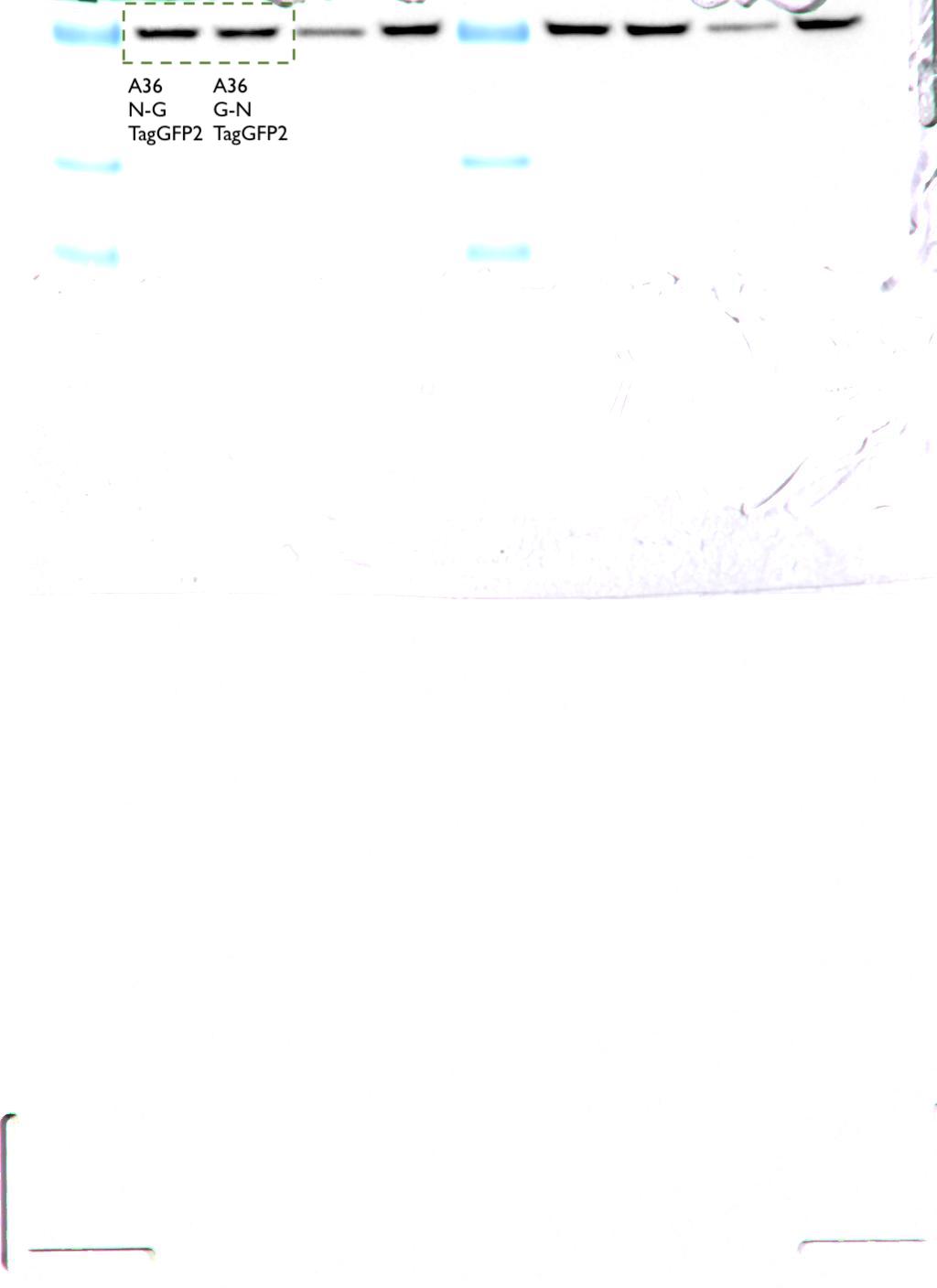

Supplement: Figure 6—figure supplement 1—source data 1. [file elife-74655-fig6-figsupp1-data1.zip › Figure 6 - supplement 1 - source data 1/Fig6-suppl1A blots/Fig6-suppl1A_Grb2 blot_annotated.jpg]

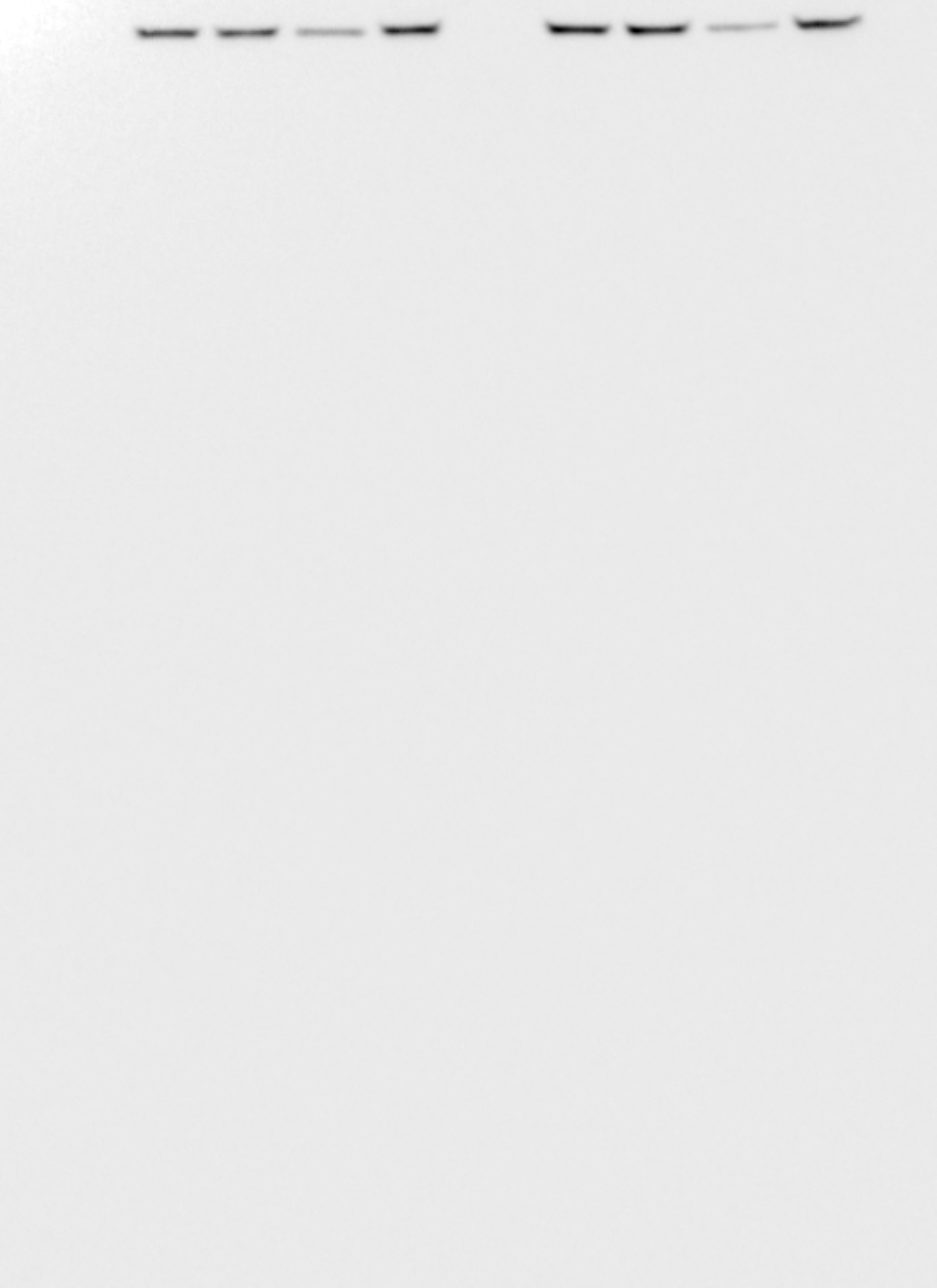

Supplement: Figure 6—figure supplement 1—source data 1. [file elife-74655-fig6-figsupp1-data1.zip › Figure 6 - supplement 1 - source data 1/Fig6-suppl1A blots/Fig6-suppl1A_Grb2 blot_raw.tif]
