# Supplementary material for: The relative binding position of Nck and Grb2 adaptors impacts actin-based motility of Vaccinia virus
Source: eLife. 2022 Jul 7;11:e74655. doi: 10.7554/eLife.74655 (PMC9333988; doi:10.7554/eLife.74655)
Supplement: Figure 4—source data 1. [file elife-74655-fig4-data1.zip › Figure 4 - source data 1/Figure 4_stats summary table.docx]

| *Figure* | *Measurement* | *Conditions* | *Test* | *p value* | *95% CI lo* | *95% CI hi* |
| --- | --- | --- | --- | --- | --- | --- |
| Fig4C | % virus w/ tails | p14 N-G vs p14 G-N | Welch’s t | 0.48204762 | -8.46 | 4.5 |
| Fig4C | Tail length | p14 N-G vs p14 G-N | Welch’s t | 0.01480858 | -1.17 | -0.21 |
| Fig4D | Virus speed | p14 N-G vs p14 G-N | Welch’s t | 0.03578749 | -0.09 | -0.01 |
| Fig4E | Plaque size | p14 N-G vs p14 G-N | Welch’s t | 0.00512664 | -0.7 | -0.26 |

* multiple comparisons tests
