# Supplementary material for: The relative binding position of Nck and Grb2 adaptors impacts actin-based motility of Vaccinia virus
Source: eLife. 2022 Jul 7;11:e74655. doi: 10.7554/eLife.74655 (PMC9333988; doi:10.7554/eLife.74655)
Supplement: Figure 5—source data 1. [file elife-74655-fig5-data1.zip › Figure 5 - source data 1/Figure 5_stats summary table.docx]

| *Figure* | *Measurement* | *Conditions* | *Test* | *p value* | *95% CI lo* | *95% CI hi* |
| --- | --- | --- | --- | --- | --- | --- |
| Fig5A | Nck intensity | A36 N-G vs A36 G-N | Welch’s t | 0.73826746 | -0.06 | 0.07 |
| Fig5A | Grb2 intensity | A36 N-G vs A36 G-N | Welch’s t | 0.0103029 | -0.14 | -0.04 |
| Fig5A | N-WASP intensity | A36 N-G vs A36 G-N | Welch’s t | 0.01907974 | -0.4 | -0.07 |
| Fig5B | Nck intensity | A36 N-G vs A36 G-N | Welch’s t | 0.86798292 | -3977.11 | 3500.43 |
| Fig5B | N-WASP intensity | A36 N-G vs A36 G-N | Welch’s t | 0.00597793 | -0.19 | -0.06 |
| Fig5C | Nck intensity | A36 N-G vs A36 G-N | Dunnett’s* | 0.8707 | -0.1244 | 0.09102 |
| Fig5C | Nck intensity | A36 N-G vs A36 N-X | Dunnett’s* | 0.9140 | -0.09436 | 0.1210 |
| Fig5C | N-WASP intensity | A36 N-G vs A36 G-N | Dunnett’s* | 0.0161 | 0.03749 | 0.2692 |
| Fig5C | N-WASP intensity | A36 N-G vs A36 N-X | Dunnett’s* | 0.1787 | -0.03917 | 0.1925 |

* multiple comparisons tests
