# Supplementary material for: The relative binding position of Nck and Grb2 adaptors impacts actin-based motility of Vaccinia virus
Source: eLife. 2022 Jul 7;11:e74655. doi: 10.7554/eLife.74655 (PMC9333988; doi:10.7554/eLife.74655)
Supplement: Figure 5—figure supplement 1—source data 1. [file elife-74655-fig5-figsupp1-data1.zip › Figure 5 - supplement 1 - source data 1/Figure 5 - supplement 1_stats summary table.docx]

| *Figure* | *Measurement* | *Conditions* | *Test* | *p value* | *95% CI lo* | *95% CI hi* |
| --- | --- | --- | --- | --- | --- | --- |
| Fig5-supp1A | RFP intensity | A36 N-G vs A36 G-N | Welch’s t | 0.82768272 | -489.48 | 429.98 |
| Fig5-supp1A | RFP intensity | GFP-Nck vs -Grb2 | Dunnett’s* | 0.5265 | -462.7 | 219.5 |
| Fig5-supp1A | RFP intensity | GFP-Nck vs -N-WASP | Dunnett’s* | 0.5998 | -234.4 | 447.9 |
| Fig5-supp1B | tgGFP2 intensity | A36 N-G vs A36 G-N | Welch’s t | 0.26334481 | -0.3 | 0.11 |
| Fig5-supp1C | WIP intensity | A36 N-G vs A36 G-N | Welch’s t | 0.11405022 | -0.45 | 0.09 |

* multiple comparisons tests
