# Supplementary material for: The relative binding position of Nck and Grb2 adaptors impacts actin-based motility of Vaccinia virus
Source: eLife. 2022 Jul 7;11:e74655. doi: 10.7554/eLife.74655 (PMC9333988; doi:10.7554/eLife.74655)
Supplement: Figure 6—source data 1. [file elife-74655-fig6-data1.zip › Figure 6 - source data 1/Figure 6_stats summary table.docx]

| *Figure* | *Measurement* | *Conditions* | *Test* | *p value* | *95% CI lo* | *95% CI hi* |
| --- | --- | --- | --- | --- | --- | --- |
| Fig6A | Src intensity | A36 N-G vs A36 G-N | Welch’s t | 0.67752574 | -0.22 | 0.17 |
| Fig6B | pY418 intensity | A36 N-G vs A36 G-N | Welch’s t | 0.61605355 | 23969.89 | -16142.32 |
| Fig6C | Nck(SH2)intensity | A36 N-G vs A36 G-N | Welch’s t | 0.80287918 | -0.36 | 0.43 |
| Fig6D | pY132 intensity | A36 N-G vs A36 G-N | Tukey’s* | 0.0025 | -74854 | -23645 |
| Fig6D | pY132 intensity | A36 N-G vs A36 N-X | Tukey’s* | 0.0496 | 48.91 | 51258 |

* multiple comparisons tests
