# Supplementary material for: The relative binding position of Nck and Grb2 adaptors impacts actin-based motility of Vaccinia virus
Source: eLife. 2022 Jul 7;11:e74655. doi: 10.7554/eLife.74655 (PMC9333988; doi:10.7554/eLife.74655)
Supplement: Figure 6—figure supplement 1—source data 1. [file elife-74655-fig6-figsupp1-data1.zip › Figure 6 - supplement 1 - source data 1/Figure 6 - supplement 1_stats summary table.docx]

| *Figure* | *Measurement* | *Conditions* | *Test* | *p value* | *95% CI lo* | *95% CI hi* |
| --- | --- | --- | --- | --- | --- | --- |
| Fig6-supp1C | pY132 intensity | AllStars vs Grb2 siRNA | Welch’s t | 0.19693593 | -27463.85 | 81286.68 |

* multiple comparisons tests
