# Supplementary material for: The relative binding position of Nck and Grb2 adaptors impacts actin-based motility of Vaccinia virus
Source: eLife. 2022 Jul 7;11:e74655. doi: 10.7554/eLife.74655 (PMC9333988; doi:10.7554/eLife.74655)
Supplement: Figure 7—source data 1. [file elife-74655-fig7-data1.zip › Figure 7 - source data 1/Figure 7_stats summary table.docx]

| *Figure* | *Measurement* | *Conditions* | *Test* | *p value* | *95% CI lo* | *95% CI hi* |
| --- | --- | --- | --- | --- | --- | --- |
| Fig7B | Tail length | A36 N-G vs A36 G-N | Tukey’s* | 0.0003 | 1.063 | 2.650 |
| Fig7B | Tail length | A36 N-G vs A36 G-N-G | Tukey’s* | 0.0067 | 0.3698 | 1.957 |
| Fig7B | Tail length | A36 N-G vs A36 G-G-N | Tukey’s* | 0.0001 | 1.316 | 2.904 |
| Fig7B | Tail length | A36 G-N vs A36 G-G-N | Tukey’s* | 0.7419 | -0.5402 | 1.047 |
| Fig7B | Tail length | A36 G-N-G vs A36 G-G-N | Tukey’s* | 0.0212 | 0.1532 | 1.740 |
| Fig7C | Virus speed | A36 N-G vs A36 G-N | Tukey’s* | 0.0015 | 0.04492 | 0.1484 |
| Fig7C | Virus speed | A36 N-G vs A36 G-N-G | Tukey’s* | 0.0082 | 0.02159 | 0.1251 |
| Fig7C | Virus speed | A36 N-G vs A36 G-G-N | Tukey’s* | 0.0006 | 0.05825 | 0.1617 |
| Fig7C | Virus speed | A36 G-N vs A36 G-G-N | Tukey’s* | 0.8412 | -0.03841 | 0.06508 |
| Fig7C | Virus speed | A36 G-N-G vs A36 G-G-N | Tukey’s* | 0.1847 | -0.01508 | 0.08841 |
| Fig7D | Plaque size | A36 N-G vs A36 G-N | Tukey’s* | 0.0027 | 0.1284 | 0.4916 |
| Fig7D | Plaque size | A36 N-G vs A36 G-N-G | Tukey’s* | 0.0788 | -0.01829 | 0.3450 |
| Fig7D | Plaque size | A36 N-G vs A36 G-G-N | Tukey’s* | 0.0035 | 0.1150 | 0.4783 |
| Fig7D | Plaque size | A36 G-N vs A36 G-G-N | Tukey’s* | 0.9951 | -0.1950 | 0.1683 |
| Fig7D | Plaque size | A36 G-N-G vs A36 G-G-N | Tukey’s* | 0.1652 | -0.04829 | 0.3150 |
| Fig7E | N-WASP intensity | A36 N-G vs A36 G-N | Tukey’s* | 0.0384 | 0.008537 | 0.2981 |
| Fig7E | N-WASP intensity | A36 N-G vs A36 G-N-G | Tukey’s* | 0.8795 | -0.1781 | 0.1115 |
| Fig7E | N-WASP intensity | A36 N-G vs A36 G-G-N | Tukey’s* | 0.0384 | 0.008537 | 0.2981 |
| Fig7E | N-WASP intensity | A36 G-N vs A36 G-G-N | Tukey’s* | >0.9999 | -0.1448 | 0.1448 |
| Fig7E | N-WASP intensity | A36 G-N-G vs A36 G-G-N | Tukey’s* | 0.0140 | 0.04187 | 0.3315 |

* multiple comparisons tests
