# Supplementary material for: The relative binding position of Nck and Grb2 adaptors impacts actin-based motility of Vaccinia virus
Source: eLife. 2022 Jul 7;11:e74655. doi: 10.7554/eLife.74655 (PMC9333988; doi:10.7554/eLife.74655)
Supplement: Figure 7—figure supplement 1—source data 1. [file elife-74655-fig7-figsupp1-data1.zip › Figure 7 - supplement 1 - source data 1/Figure 7 - supplement 1_stats summary table.docx]

| *Figure* | *Measurement* | *Conditions* | *Test* | *p value* | *95% CI lo* | *95% CI hi* |
| --- | --- | --- | --- | --- | --- | --- |
| Fig7-supp1A | % virus w/ tails | A36 N-G vs A36 G-N | Tukey’s* | 0.6528 | -8.371 | 18.19 |
| Fig7-supp1A | % virus w/ tails | A36 N-G vs A36 G-N-G | Tukey’s* | 0.7972 | -9.479 | 17.08 |
| Fig7-supp1A | % virus w/ tails | A36 N-G vs A36 G-G-N | Tukey’s* | 0.3775 | -6.179 | 20.38 |
| Fig7-supp1A | % virus w/ tails | A36 G-N vs A36 G-G-N | Tukey’s* | 0.9497 | -11.09 | 15.47 |
| Fig7-supp1A | % virus w/ tails | A36 G-N-G vs A36 G-G-N | Tukey’s* | 0.8544 | -9.979 | 16.58 |
| Fig7-supp1B | Nck intensity | A36 N-G vs A36 G-N | Tukey’s* | 0.9731 | -0.1432 | 0.1099 |
| Fig7-supp1B | Nck intensity | A36 N-G vs A36 G-N-G | Tukey’s* | 0.8703 | -0.09653 | 0.1565 |
| Fig7-supp1B | Nck intensity | A36 N-G vs A36 G-G-N | Tukey’s* | 0.7913 | -0.08986 | 0.1632 |
| Fig7-supp1B | Nck intensity | A36 G-N vs A36 G-G-N | Tukey’s* | 0.5602 | -0.07319 | 0.1799 |
| Fig7-supp1B | Nck intensity | A36 G-N-G vs A36 G-G-N | Tukey’s* | 0.9981 | -0.1199 | 0.1332 |
| Fig7-supp1C | Grb2 intensity | A36 N-G vs A36 G-N | Tukey’s* | 0.0024 | 0.1412 | 0.5255 |
| Fig7-supp1C | Grb2 intensity | A36 N-G vs A36 G-N-G | Tukey’s* | 0.1968 | -0.3255 | 0.05881 |
| Fig7-supp1C | Grb2 intensity | A36 N-G vs A36 G-G-N | Tukey’s* | 0.0024 | 0.1412 | 0.5255 |
| Fig7-supp1C | Grb2 intensity | A36 G-N vs A36 G-G-N | Tukey’s* | >0.9999 | -0.1921 | 0.1921 |
| Fig7-supp1C | Grb2 intensity | A36 G-N-G vs A36 G-G-N | Tukey’s* | 0.0002 | 0.2745 | 0.6588 |

* multiple comparisons tests
