# Supplementary material for: The relative binding position of Nck and Grb2 adaptors impacts actin-based motility of Vaccinia virus
Source: eLife. 2022 Jul 7;11:e74655. doi: 10.7554/eLife.74655 (PMC9333988; doi:10.7554/eLife.74655)
Supplement: Figure 7—figure supplement 2—source data 1. [file elife-74655-fig7-figsupp2-data1.zip › Figure 7 - supplement 2 - source data 1/Figure 7 - supplement 2_stats summary table.docx]

| *Figure* | *Measurement* | *Conditions* | *Test* | *p value* | *95% CI lo* | *95% CI hi* |
| --- | --- | --- | --- | --- | --- | --- |
| Fig7-supp2A | Plaque size | A36 G-N-G clone 1 vs 2 | Welch’s t | 0.90641681 | -0.67 | 0.61 |
| Fig7-supp2B | Plaque size | A36 G-G-N clone 1 vs 2 | Welch’s t | 0.49468885 | -0.25 | 0.44 |

* multiple comparisons tests
